# TRPV6 channel function is involved in endometrial epithelial cell Ca^2+^ signaling and female mouse fecundity

**DOI:** 10.1007/s00018-025-05857-9

**Published:** 2025-10-07

**Authors:** Adela Sota, Andreas Beck, Philipp Wartenberg, Anna-Lena Gehl, Manuel Winter, Ulrich Wissenbach, Marc Freichel, Markus R. Meyer, Ulrich Boehm, Veit Flockerzi, Claudia Fecher-Trost, Petra Weissgerber

**Affiliations:** 1 Experimental and Clinical Pharmacology and Toxicology, Center for Molecular Signaling (PZMS), Saarland University, 66421 Homburg, Germany; 2 Center for Gender-Specific Biology and Medicine (CGBM), Saarland University, 66421 Homburg, Germany; 3 Institute of Pharmacology, Heidelberg University, 69120 Heidelberg, Germany

**Keywords:** Transient receptor potential vanilloid 6, Endometrium, Epithelium, Cytosolic Ca^2+^ imaging, Whole-cell patch clamp

## Abstract

The Ca^2+^-selective transient receptor potential vanilloid 6 (TRPV6) channel plays a fundamental role in the female and male murine reproductive system. We have previously shown that TRPV6 is essential for male fertility, and necessary for a proper placental Ca^2+^ transport, embryonic bone development and calcification, as well as for extracellular matrix formation in the placental labyrinth. Here, we show that lack of functional TRPV6 results in impaired fecundity in female mice with increased latency to first pregnancy, longer interpregnancy intervals and fewer and smaller litters. In mouse endometrium the TRPV6 protein is expressed in epithelial cells (MEECs). Using patch clamp recording and Ca^2+^ imaging, we show TRPV6-dependent whole-cell currents and that TRPV6 contributes to cytoplasmic Ca^2+^ signaling in MEECs. MEECs lacking functional TRPV6 Ca^2+^ channels reveal a significantly reduced frequency of spontaneous cytosolic Ca^2+^ oscillations, shown in isolated cells and in situ in whole mount uterus preparations. Our results reveal a previously unknown physiological role for TRPV6 in the regulation of endometrial Ca^2+^ homeostasis and its impact on female fecundity in mice, providing a molecular and cellular framework for further investigation of reproductive disorders, such as those associated with defective Ca^2+^ regulation in women.

**Supplementary Information:**

The online version contains supplementary material available at 10.1007/s00018-025-05857-9.

## Introduction

It is well known that Ca^2+^ plays a major role in many stages of the reproductive process, from germ cell maturation to placental and embryonic development. However, the precise function and regulation of Ca^2+^ during subsequent reproductive processes is incompletely understood.

The transient receptor potential (TRP) vanilloid 6 (TRPV6) channel is highly selective for Ca^2+^ and plays a fundamental role for Ca^2+^ (re)-uptake [1, 2] and transcellular Ca^2+^ transport across epithelial tissue barriers [3–5]. Thus, TRPV6-dependent cytosolic Ca^2+^ changes initiate and coordinate different signaling pathways and thereby cellular and systemic physiological and pathophysiological processes [6]. In the last years, remarkable progress has been made in structural analysis of TRPV6 in both closed and open channel states [7, 8]. Structures have been revealed in the absence and presence of divers modulators such as 2-APB, (4-phenylcyclohexyl) piperazine derivatives (PCHPDs) such as cis-22a, ruthenium red and econazole, or genistein [9–17]. However, pharmacological tools remain limited and are not yet specifically targeted at TRPV6 function in isolated primary cells, nor do they appear to be particularly effective, as with soricidin [18].

In mice, TRPV6 is expressed in exocrine pancreas, salivary gland, placenta, small intestine, cecum, prostate and epididymis [2, 3, 6, 19–22]. Lack of *Trpv6* results in hypofertility of male mice [1, 2]. A decreasing intraluminal Ca^2+^ concentration along the epididymal segments is essential to produce mature spermatozoa during the epididymal passage. Using *Trpv6*-deficient (*Trpv6*^−/−^) mice and mice carrying a single-point mutation within the channel pore of TRPV6 (D541A, new nomenclature D581A [23]; *Trpv6*^*mt/mt*^), leading to a non-functional TRPV6 channel [2], we showed that TRPV6 proteins are essential constituents of the underlying Ca^2+^ uptake mechanism in the epididymis. In female mice, TRPV6 is expressed in the yolk sac and in labyrinth trophoblasts of the placenta contributing to maternal-fetal Ca^2+^ supply of the embryo. The absence of the channel leads to impaired bone growth with shorter and less calcified femurs in the offspring, given that *Trpv6*-deficient trophoblasts absorb significantly less Ca^2+^ from the maternal blood [3]. In human, TRPV6 loss-of-function mutations may also result in under-mineralized bones and skeletal dysplasia with postnatal recovery [24, 25] and transient neonatal hyperparathyroidism [26–28].

Embryo transfer experiments revealed that both the maternal and fetal parts of the placenta contribute to embryonic development and Ca^2+^ accumulation in the bones [3]. In addition to expression in the fetal structures, i.e. placental labyrinth and yolk sac, TRPV6 is also expressed in the maternal part of the placenta, the decidua [3]. The decidua is formed by a transformation of the maternal endometrium, a process named decidualization and is the essential prerequisite for both the implantation of the blastocyst and the maintenance of the pregnancy. This suggests that the *Trpv6*-deficient phenotype of the embryo is probably not only caused by the lack of TRPV6 in trophoblasts but also depends on its presence and probably channel function in the endometrium.

A successful pregnancy requires a complex dialogue between the implanting embryo and the endometrium. The multi-step process of embryo implantation is initiated by the hatched blastocyst and followed by adhesion, attachment and subsequent invasion of trophoblast cells through the endometrial epithelium into the stroma [29, 30]. The human endometrium constitutes the inner lining of the uterus and undergoes monthly cycles of breakdown and repair in preparation for a possible pregnancy. It consists of a single layer of epithelial cells lining the uterine lumen and the underlying stroma which varies in thickness according to fluctuations of the ovarian hormones estrogen and progesterone. While decidualization in humans routinely occurs during the monthly estrous cycle and is propagated by the invasion of a blastocyst, the initiation of decidualization in mice requires the presence of the blastocyst in the uterine lumen [30–32]. However, the exact mechanism of decidualization, the exact signaling between the endometrial epithelial cells, which get in contact with the blastocyst, and the underlying endometrial stromal cells, which then proliferate and differentiate into decidual cells, supporting the implantation of the embryo, remain poorly understood. The need for Ca^2+^ in the different gestational processes implicates the presence of specialized ion channels to regulate Ca^2+^ homeostasis [29]. The TRPV6 channel might be the sensor and messenger molecule in the endometrial epithelial cells involved in the transformation of extracellular stimuli into the influx of Ca^2^, inducing and coordinating underlying signaling pathways.

Using isolated uteri and primary endometrial cells from wild-type mice, we identified an almost exclusive expression of TRPV6 in the epithelial cells of the murine endometrium. Furthermore, our studies uncovered that TRPV6 channels contribute to Ca^2+^ influx and spontaneous Ca^2+^ oscillations in the wild-type mouse endometrial epithelial cells (MEECs), in which we recorded distinct TRPV6-dependent whole-cell currents. Phenotypically, female *Trpv6*^*−/−*^ [1] and *Trpv6*^*mt/mt*^ mice [2], both lacking functional TRPV6 channels, revealed increased latency to first pregnancy, longer interpregnancy intervals and fewer and smaller litters. Our results suggest, that TRPV6-dependent Ca^2+^ influx in MEECs contributes to the decidualization process and thus to female fecundity in mice.

## Materials and methods

### Mice

All animal care and experimental procedures were reviewed and approved in accordance with the guidelines and ethical regulations established by the animal welfare committee of Saarland University. Adult (8–12 weeks old) female mice were kept under a standard light/dark cycle (12 h/12 h) with food and water *ad libitum*.

Studies were performed on wild-type mice of the mixed 129/SvJ × C57BI/6 N background, which is the background of the *Trpv6*^*−/−*^, *Trpv6*^*mt/mt*^, *Trpv6*-IC and *Trpv6*^*−/−*^-IC mice (see below). *Trpv6*^*−/−*^ mice carry a deletion of about one third of the protein coding region of the *Trpv6* gene including exons 13, 14 and 15, coding for a part of domain S5, the channel pore, domain S6, and the cytosolic C-terminus [1]. The *Trpv6*^*mt/mt*^ mice represent a functional TRPV6 knock-out, homozygously carrying a single-point mutation within exon 13, coding for the channel pore (D541A, new nomenclature D581A [23]), leading to a non-functional TRPV6 channel [2].

To visualize TRPV6-expressing cells, *Trpv6*-IRES-Cre mice (*Trpv6*-IC; [3]) were bred to homozygous enhanced-Rosa26-floxed-stop-reporter mice (eR26-τGFP; [33]). Due to a loxP flanked (floxed) strong transcriptional termination sequence, the eR26-reporter allele terminates τGFP transcription prematurely, but when the mice are crossed with Cre-expressing mice, the Cre-mediated excision of the floxed termination sequence leads to constitutive τGFP expression. All *Trpv6*-IC/eR26-τGFP animals in the F1 generation are heterozygous for the *Trpv6*-IC and eR26-τGFP alleles and exhibit τGFP exclusively in *Trpv6*-expressing cells, more precisely in cells where the TRPV6 promotor had been active [34].

To visualize and to analyze cells, in which the TRPV6 gene has been knocked-out, we generated a new *Trpv6*^*−/−*^-IRES-Cre (*Trpv6* KO-IC) mouse strain (see Fig. S3). For construction of the targeting vector (LpmCaTL_88), genomic DNA was isolated from R1 ES cells and used as a template for polymerase chain reaction (PCR) amplification of 5′ and 3′ homology arms with *Pfu* polymerase. The genomic sequence of the 5′ homology contained exons 6 to 12 of the *Trpv6* gene and 3 additional stop codons in 3 different reading frames and a DTA cassette for negative selection. An internal ribosome entry site (IRES) sequence followed by a Cre recombinase complementary DNA was inserted after the final stop codon. The IRES element will result in the production of a bicistronic messenger RNA, from which TRPV6 and Cre recombinase are independently translated. This sequence is followed by an FRT (Flp recognition target) sequence-flanked pgk-promotor-driven neomycin resistance gene cassette (neo^r^) and a Flp-ACE cassette, which directs self-induced deletion of DNA sequences as they pass through the male germ line [35]. The testes-specific promoter from the angiotensin-converting enzyme gene (ACE) was used to drive the expression of the Flp-recombinase gene. The 3′ homology arm was cloned downstream of this cassette. An enhanced GFP cassette and the herpes simplex virus thymidine kinase (tk) cassette were introduced for negative selection (Fig. S3a). ES cell culture was essentially done as described [2, 36]. 10 of 333 double-resistant, GFP-negative colonies showed correct homologous recombination at the *Trpv6* locus (*Trpv*6L2). GFP-positive cell colonies were discarded. Recombination was confirmed by Southern Blot hybridization with a 5′ and 3′ probe external to the targeting vector and a neo probe (Fig. S3b). Germline chimeras were obtained by injection of 2 selected ES cell clones into C57Bl/6 blastocysts and subsequently crossed with C57Bl6/N mice to get animals heterozygous for the *Trpv6*^*−/−*^-IC allele where the neo cassette is already removed (Fig. S3c, d). *Trpv6*^−/−^-IC mice were kept on a mixed (129/SvJ × C57BI/6 N) background and bred to homozygosity. To visualize cells in which the TRPV6 gene has been knocked out, two generations of breeding are needed. The first breeding consists of a cross between the *Trpv6*^*−/−*^-IC and eR26-τGFP mice. The F1 *Trpv6*^*−/−*^-IC/eR26-τGFP mice are heterozygous for both *Trpv6*^*−/−*^-IC and eR26-τGFP alleles. Now, female F1 mice are bred with male heterozygous *Trpv6*^*−/−*^-IC mice to produce the target genotype (note: homozygous male *Trpv6*^*−/−*^ mice are hypofertile [1]. This drastically lowers the probability of producing the target genotype to only 6.25% of the offspring according to Mendel. However, the actual frequency of the desired genotype in females was only 5.43%. Taking into account that the average litter size was 6.93 ± 1.12 (81 litters from 23 breeding pairs) extensive breeding efforts are required to obtain a small number of female animals with the correct genotype and age. All female mice finally used were homozygous for the *Trpv6*^−/−^-IC allele and heterozygous for the eR26-τGFP allele and express τGFP in cells where the *Trpv6* promotor had been active.

To analyze cytosolic Ca^2+^ changes in TRPV6-expressing cells in isolated uteri in situ, *Trpv6*-IC mice were crossed with eR26-GCaMP3 mice, having the calcium indicator GCaMP3 inserted into the Rosa26 locus [37]. A loxP-flanked triple stop signal blocks the expression of GCaMP3. The F1 *Trpv6*-IC/eR26-GCaMP3 mice are heterozygous for both *Trpv6*-IC and for eR26-GCaMP3 alleles and exclusively exhibit the GCaMP3 Ca^2+^-sensor protein in *Trpv6*-expressing cells. To analyze cytosolic Ca^2+^ changes in TRPV6 knock-out cells in isolated uteri in situ again two generations of breeding are needed. The first breeding consists of a cross between the *Trpv6*^*−/−*^-IC and eR26-GCaMP3 mice. The F1 *Trpv6*^*−/−*^-IC/eR26-τGFP mice are heterozygous for both *Trpv6*^*−/−*^-IC and eR26-τGFP alleles (Fig. S3e). Now, female F1 mice are bred with male heterozygous *Trpv6*^*−/−*^-IC mice to produce the target genotype. All female animals finally used were homozygous for the *Trpv6*^−/−^-IC allele and heterozygous for the eR26-GCaMP3 allele, expressing GCaMP3 only in cells where the *Trpv6* promotor had been active.

The *Trpv6*- and *Trpv6*^*−/−*^-IC/eR26-τGFP mice were used to analyze the TRPV6 expression profile in the endometrium, and the *Trpv6*- and *Trpv6*^*−/−*^-IC/eR26-GCaMP3 mice served for the functional analysis (cytosolic Ca^2+^ imaging) of TRPV6 channels in isolated uteri in situ.

### Fecundity analysis

Fecundity is defined as the ability to reproduce, i.e. to produce offspring, in contrast to fertility, which indicates the ability to conceive. To quantify the fecundity rate of wild-type, *Trpv6*^*−/−*^ and *Trpv6*^*mt/mt*^ mice, we analyzed and averaged the litter size and the time interval between subsequent litters for each mating couple over a period of up to 12 months and calculated the ratio from both. *Trpv6*^*−/−*^ and *Trpv6*^*mt/mt*^ mating couples comprised of heterozygous male and homozygous female mice.

### Vaginal cytology and standardization of estrous cycle stage

Estrous cycle stages of the female mice were identified by vaginal cytology [38, 39]. Vaginal lining was flushed 3–5 times with 40 µL NaCl and the final cell suspension was placed on a glass slide and examined under a bright field light microscope using a 10X objective (Zeiss Axio Imager.M2, Carl Zeiss, Oberkochen, Germany). *Trpv6* expression and proliferation of endometrial cells in mice is highest at estrus (Fig. 2 and [40]). Thus, only mice that were in estrus, identified by the dominant presence of cornified epithelial cells and the lack of leukocytes in the vaginal smear, had been used for further experiments. To standardize the estrous cycle stage, adult female mice were injected subcutaneously with 50 µL of 17β-estradiol (E2) solution (100 ng/50 µL sesame oil; Sigma-Aldrich, St. Louis and Burlington, MA, USA) for three consecutive days prior to uterus and cell isolation.

### Hormone measurements

Trunk blood was collected from *Trpv6*^*−/−*^ and control mice, allowed to clot for 30 min at room temperature, centrifuged for 10 min at 4 °C at 2,000xg, the serum removed and subsequently centrifuged for a further 10 min at 4 °C at 2,000xg. The serum was stored at −20 °C until analyzed. Hormone measurements were performed using the Luminex xMAP technology (MAGPIX, Luminex Corporation) in combination with the mouse pituitary kit (MPTMAG-49 K, Merck Millipore) according to the manufacturer’s instructions and analyzed with the xPONENT software (Luminex Corporation) according to the protocol (Luminex/MILLIPLEX MAP Human Pituitary Magnetic Bead Panel, Merck). Samples were pipetted as duplicates and the mean was calculated. Measurements with an intra-assay value above 20% were excluded. In four independent runs the quality controls were within 97% of the expected range. 3% were below the minimum expected value.

### Primary cells isolation

The isolation of mouse endometrial epithelial cells (MEECs) and stromal cells (MESCs) was performed according to the method described by De Clercq et al. [40]. Uterine horns were dissected and placed in a dish containing Hanks’ Balanced Salt Solution (HBSS+, Thermo Fisher Scientific, Waltham, MA, USA) supplemented with 100 U/mL penicillin and 100 µg/mL streptomycin. All residual adipose and connective tissue were removed under the stereo microscope (Zeiss Stemi 2000-CS). Uterine horns were cut open longitudinally to expose the uterine lumen and transferred to a tube containing 2.5% pancreatin and 0.25% trypsin in HBSS+. The tube was incubated horizontally for 60 min at 4 °C on a shaker, 45 min at room temperature (RT, no shaking) and 15 min at 37 °C in a water bath (no shaking). The following MEEC and MESC isolation steps were performed in a sterile environment under a laminar flow cabinet.

For MEEC isolation, after two hours of incubation, the uteri were transferred into a dish with cold MEEC medium (DMEM, Sigma) containing 10% FBS (Thermo Fisher Scientific), 0.5 µg/mL amphotericin B (Thermo Fisher Scientific), 100 µg/mL gentamicin (Thermo Fisher Scientific), 25% MCDB-105 medium (Cell Applications Inc, San Diego, CA, USA), 5 µg/mL insulin (Sigma)) for 5 min to inactivate trypsin activity. The digested tissue was then transferred into a tube containing cold HBSS+. The tube was vortexed for 10 s to release the epithelial sheets and the tissue was rinsed in a clean Petri dish with 3 mL HBSS + and vortexed in two additional tubes, obtaining a total of three tubes containing epithelial sheets. The epithelial sheets were recovered by gently pipetting the three cell suspensions on a 100 μm nylon mesh to remove tissue debris. The collected cell suspension was centrifuged at 500 x g for 5 min. The pellet was resuspended in 12 mL of MEEC medium and mixed well. The solution was put aside to settle for 5 min in order to separate remaining MESC by gravity sedimentation. After 5 min, the upper 2 mL of the suspension were removed and the cell suspension was centrifuged again at 500 x g for 5 min and resuspended in 3 mL MEEC medium, depending on the number of isolated uteri and the finally desired experimental cell density.

The MESCs were then isolated from the same preparation according to the protocol [40]. Therefore, two digestion mixtures were prepared by dissolving 300 µL of the 1 mg/mL collagenase (Sigma) in 2.7 mL of 0.05% trypsin/EDTA solution (Thermo Fisher Scientific). Three small Petri dishes were filled with cold (4 °C) HBSS + and 3 × 15 mL tubes with 3 mL of MESC medium. After 30 min of incubation in the first MESC digestion mix, the digested tissue trypsin solution was shaken gently for 10 s to detach the MESCs from the uterine tissue. The uteri were then transferred into the first Petri dish containing 3 mL cold (4 °C) HBSS + and rinsed well. 3 mL of the MESC medium were added into the MESC digestion mix to inhibit trypsin activity. After rinsing in HBSS+, the uteri were transferred to one of the tubes containing 3 mL of MESC medium and shaken gently for 10 s. This step was repeated three times in total (transferring uteri from HBSS + to MESC medium), so the uteri were rinsed and gently shaken for 10 s in each of the three tubes. In the end, this protocol results in four MESC suspensions: one tube containing MESCs in trypsin solution and MESC medium, and three tubes with MESCs in MESC medium. Finally, the uteri were transferred in the second MESC digestion mix and incubated in a water bath for 30 min at 37 °C, vertically. Yet, the collected cells are an impure collection of mostly stromal cells and some epithelial cells. Therefore, another three small Petri dishes were prepared with cold (4 °C) HBSS + and 3 × 15 mL tubes with 3 mL of MESC medium. After the uteri were shaken gently for 10 s in four separate tubes, the stromal cells were collected by passing the content of the tubes through a 40 μm nylon mesh. The mesh was rinsed with an additional 5 mL of MESC medium. The cell suspension was centrifuged at 500 x g for 7 min and the pellet was resuspended in 3 ml MESCs medium.

Finally, almost pure cultures of MEECs and MESCs from wild-type, *Trpv6*^*−/−*^ and *Trpv6*^*mt/mt*^ mice were obtained and plated on 12 mm and 25 mm collagen-coated coverslips and incubated at 37 °C with 5% CO_2_ in preparation for biochemical and functional experiments. Apparently, both primary cell types, isolated from the three genotypes (wild-type, *Trpv6*^*−/−*^ and *Trpv6*^*mt/mt*^), revealed no morphological differences in culture.

### Histological staining

24 h after plating, the MEECs and MESCs were fixed with cold absolute ethanol for 5 to 7 min and then gently rinsed in cold tap water to wash and hydrate the cells. After bathing in hematoxylin (Morphisto GmbH, Offenbach am Main, Germany) for 6 min and in warm tap water for 4 min, cells were incubated in eosin (Mephisto GmbH) for another 6 min and quickly rinsed in tap water before going through a series of dehydration. The cells were dipped in two different concentrations of ethanol (70%, 96%), then washed in absolute ethanol for 2 min and finally incubated in xylene (Applied Biosystems, Waltham, MA, USA) for clearing. After 3 min, the cells were mounted by using a non-aqueous mounting medium (Depex; Serva, Heidelberg, Germany) and imaged with an automated slide scanner (Zeiss Axio Scan Z1).

### Immunohistochemistry

MEEC and MESC cultures were washed 3 × 5 min with PBS on a shaker, fixed for 10 min with 4% paraformaldehyde (PFA, Sigma; no shaking) and washed three times with PBS without Ca^2+^ and Mg^2+^ (Thermo Fisher Scientific). To stain cytokeratin and vimentin, established marker proteins of MEECs and MESCs, respectively [40, 41], the cells were permeabilized under shaking (50 rpm) with 0.2% Triton-X 100 (Carl Roth, Karlsruhe, Germany) for 10 min and washed again three times with PBS before they were incubated for 2 h in 5% normal goat serum (NGS; Vector Laboratories, Newark, CA, USA) in PBS to block non-specific antibody binding. Finally, MEECs and MESCs were stained with antibodies against established markers, cytokeratin (1:1000, Sigma, MEECs) and vimentin (1:500, Cell Signaling Technology, Danvers, MA, USA, MESCs) respectively at 4 °C on a shaker according to [40]. All antibodies were diluted in PBS with 0.5% NGS. After 24 h of incubation in the primary antibody solution, the cells were first washed three times with PBS on a shaker, then they were incubated in the dark with secondary antibodies (Alexa Fluor 594-conjugated goat anti-mouse IgG (Invitrogen) and Alexa Fluor 488-conjugated goat anti-rabbit IgG (Invitrogen); 1:1000 in 0.5% NGS) for 1 h on a shaker. They were washed another three times with PBS on a shaker and then incubated in the dark for nuclear staining with Hoechst 33258 (1:1000, Sigma) in PBS for 15 min. After a final triple wash with PBS, the coverslips were mounted on glass slides (Fluoromount, Southern Biotech). For GFP staining, the cells were permeabilized and blocked for 1 h at RT using a blocking solution containing 0.2% Triton X-100 and 5% normal donkey serum (NDS, Jackson Immuno Research). MEECs and MESCs were then incubated overnight at 4 °C with primary antibodies; MEECs were incubated with monoclonal mouse anti-pancytokeratin (1:1000, Sigma) and chicken anti-GFP (1:1000, Thermo Fisher Scientific), and MESCs were incubated with monoclonal rabbit anti-vimentin (1:500, Cell Signaling Technology) and chicken anti-GFP. Antibodies were diluted in the blocking solution. Cells were washed the next day 3 × 5 min with PBST (0.05% Tween 20 in PBS) and incubated with secondary antibodies for 2 h at RT; MEECs were incubated with anti-mouse Cy5 (1:1000, company) and Alexa Fluor 488 donkey anti-chicken IgG (1:500, Jackson Immuno Research), and MESCs were incubated with anti-rabbit Cy5 (1:1000, Jackson Immuno Research) and Alexa Fluor 488 donkey anti-chicken IgG (1:500, Jackson Immuno Research). The secondary antibodies were diluted in PBS. The cells were washed 3 × 5 min with PBST and incubated with Hoechst 33258 (1:1000) in PBS for 10 min in the dark at RT. The cell-containing coverslips were finally washed 2 × 5 min with PBST and mounted upside down on a slide with Fluoromount (Southern Biotech). All slides were imaged using an epifluorescence microscope (Zeiss Axio Imager M2).

### Immunolabeling-enabled three-dimensional imaging of solvent-cleared organs (iDISCO)

Adult (animal number, *n* = 3 for *Trpv6*-IC/eR26-τGFP, *n* = 5 for *Trpv6*^*−/−*^-IC/eR26-τGFP) female mice were anesthetized with a mix of ketamine and xylazine. Mice were transcardially perfused with PBS, followed by 4% paraformaldehyde (PFA). The uteri and ovaries were dissected and post-fixed in 4% PFA for 3 h at 4 °C. The samples were then slowly dehydrated at room temperature (RT) in increasing concentrations of methanol (VWR Chemicals, Radnor, PA, USA). Dehydration was followed by overnight delipidation in a 66% dichloromethane (DCM, Sigma)/33% methanol solution at 4 °C with rotation. The uteri were then washed in methanol in RT, chilled at 4 °C for 2 h, and bleached in 5% hydrogen peroxide (H_2_O_2_, Sigma). Each sample was rehydrated the next day in a series of decreasing concentrations of methanol. This was followed by incubation in a blocking and permeabilizing solution (PBSGT: 1X PBS, 0.2% Gelatin (VWR Chemicals), 1% Triton X-100 and 0.02% sodium azide (Sigma) against microbial contamination) for 4 days with rotation at RT. All the following antibody incubation steps were performed at 37 °C to increase antibody penetration. Samples were first incubated with primary antibodies (rabbit anti-GFP, 1:5000, Invitrogen) in PBSGT for 2 weeks with rotation. After the incubation, they were washed several times during the course of the day as well as overnight. Samples were incubated with secondary antibodies (donkey anti-rabbit Cy5, 1:1000, Jackson ImmunoResearch Inc., West Baltimore Pike, PA, USA) in PBSGT for 1 week with rotation. All steps following incubation with secondary antibodies were performed in dark conditions. They were further incubated in increasing concentrations of methanol and then delipidized overnight. Next, the uteri were incubated in 100% DCM with rotation until they sank at the bottom of the container, then they were transferred in 100% benzyl ether (DBE; Sigma). After 2 h of clearing, samples were stored in a new DBE solution in the dark [42]. The tissues were imaged using a light-sheet microscope (UltraMicroscope Blaze™, Miltenyi Biotec, Bergisch Gladbach, Germany).

### Mass spectrometry (MS)

#### TRPV6 immunoprecipitations from mouse uterus, MEECs and MESCs

Uteri from different genotypes and estrous stages or MEEC and MESC cells were resuspended in RIPA buffer (150 mM NaCl, 50 mM Tris HCl, pH 8.0, 5 mM EDTA, 1% Nonidet P40, 0.1% SDS, 0.5% Na-deoxycholate, pH 7.4), supplemented with proteinase inhibitors (Roche, Mannheim, Germany). Uteri tissue was minzed by ultraturrax treatment or MESC and MEEC cell solution was sheared ten times (27G gauge needle) on ice and then incubated for 30 min at 4 °C on a shaker. After centrifugation at 100,000x g at 4 °C for 45 min, the supernatant containing the solubilized proteins was collected and the protein concentration was determined by Biochinonic BCA-assay (Thermo Fisher Scientific, Germany). 10 mg uterine proteins or 0.6–1.2 mg MESC/MEEC proteins were incubated for 16 h at 4 °C in the presence of 10 µg anti-TRPV6 antibody 1271 (directed against the N-terminus) or anti-TRPV6 antibody 429 (directed against the C-Terminus) coupled to 50 µl of Dynabeads™Protein G (Invitrogen, Schwerte, Germany). The beads were collected using a magnetic rack, washed three times with 1 mL RIPA buffer and were eluted with 50 µL denaturing sample buffer (final concentration: 60 mM Tris HCl, pH 6.8, 4% SDS, 10% glycerol including 0.72 M β-mercaptoethanol). The elute was incubated for 20 min at 60 °C and analysed by mass spectrometry. The same elutes were used for western blot analysis (Figs. 2 and 3d). For TRPV6 detection, membranes were incubated with the monoclonal C-terminal TRPV6 antibody (20C6).

#### Gel electrophoresis of proteins and sample Preparation for mass spectrometry

Proteins elutes from the TRPV6 IPs were separated on NuPAGE^®^ 4%−12% Bis-Tris gradient gels (Thermo Fisher Scientific, Germany), fixed in the presence of 40% ethanol and 10% acetic acid, incubated 3 times for 10 min with water and stained with Coomassie (0.12% (w/v) Coomassie G-250 (20% (v/v) methanol, 10% (v/v) phosphoric acid, 10% (w/v) ammonium sulfate). Stained gel areas were cut in pieces and washed twice alternately with buffer A (50 mM NH_4_HCO_3_) and buffer B (50 mM NH_4_HCO_3_/50% (v/v) acetonitrile). Reduction of disulfide bonds was done by incubation at 56 °C for 30 min in the presence of 10 mM dithiothreitol (Applichem, Germany) in buffer A, followed by carbamidomethylation with iodacetamide (Thermo Scientific, Germany) at 21 °C in darkness for 30 min in the presence of 5 mM iodoacetamide in buffer A. Gel pieces were washed twice alternating with buffer A and B and then dried in a vacuum centrifuge. For in-gel digestion, the gel pieces were incubated in the presence of 15 µL of porcine trypsin (10 ng/µl, Promega) in buffer A at 37 °C overnight. Tryptic peptides were extracted twice with 50 µL extraction buffer (2.5% formic acid/50% acetonitrile) in an ultrasonic bath. Both supernatants were combined and concentrated in a vacuum centrifuge and resuspended finally in 21 µL of 0.1% formic acid.

#### Nano ESI-LC-MS^2^ measurements

Tryptic peptides were analysed by nanoflow LC-HR-MS/MS (Ultimate 3000 RSLC nano UHPLC-system coupled to an LTQ Orbitrap Velos Pro or an Eclipse Tribrid mass spectrometer (all Thermo Fisher Scientific, Germany). Peptides analysed by the Orbitrap Velos setup were first trapped on a column (100 μm x 2 cm, Acclaim PepMap100C18, 5 μm, Thermo Fisher Scientific) and separated on a reversed phase C18 column (Acclaim PepMap capillary column, C18; 2 μm; 75 μm x 25 cm, Thermo Fisher Scientific) at a flow rate of 200 nL/min during a 120 min gradient build with buffer A (water and 0.1% formic acid) and B (90% acetonitrile and 0.1% formic acid). Eluted peptides were directly sprayed into the mass spectrometer through a coated silica electrospray emitter (PicoTipEmitter, 30 μm, New Objective) and ionized at 2.2 kV. MS spectra were acquired in a data-dependent mode. Full scan MS spectra (m/z 300–1700) were acquired in the Orbitrap analyser using a target value of 10e^6^. The 10 most intense peptide ions with charge states > + 2 were fragmented in the high-pressure linear ion trap by low-energy CID (35% normalized collision energy). Peptides analysed with an Eclipse Tribrid mass spectrometer (Thermo Scientific, Germany) were first trapped on a C18 trap column (75 μm × 2 cm, Acclaim PepMap100C18, 3 μm, nano viper) and separated on a reverse phase column (nano viper Acclaim PepMap column, C18; 2 μm; 75 μm × 50 cm). Peptides were separated for 120 min by a gradient, generated with buffer A and buffer B at a flow rate of 300 nl/min. The effluent was sprayed into an Orbitrap Eclipse Tribrid mass spectrometer (Thermo Scientific, Germany) using a coated emitter (PicoTipEmitter, 30 μm, New Objective, Woburn, MA, USA, ionization energy: 2.4 keV) and measured in data dependent mode. MS^1^ peptide spectra were acquired using the Orbitrap analyzer (*R* = 120k, RF lens = 30% m/z = 375–1500, MaxIT: auto, profile data, intensity threshold of 10e^4^). Dynamic exclusion of the 10 most abundant peptides was performed for 60 s. MS^2^ spectra were collected in the linear ion trap (isolation mode: quadrupole, isolation window: 1.2, activation: HCD, HCD collision energy: 30%, scan rate: fast, data type: centroid).

#### Raw LC-MS^2^ data analysis

Tryptic peptides were identified by analysing raw data with the MASCOT algorithm and Proteome Discoverer 1.4 (Thermo Fisher Scientific, Germany) software or Peaks Studio10.6 (Bioinformatic Solutions Inc. Canada). Peptides were matched to tandem mass spectra by Mascot version 2.4.0 (Matrix Science) by searching an SwissProt database (version 2018_05, number of protein sequences 557.992 containing 16.992 mus musculus sequences) against mouse proteins. MS^2^ spectra were matched with a mass tolerance of 7 ppm for precursor masses and 0.5 Da for peptide fragment ions. Tryptic digest, two missed cleavage sites, cysteine carbamidomethylation as a fixed modification and deamidation of asparagine and glutamine, acetylation of lysine and oxidation of methionine as variable modifications were used for the search. The MASCOT output files were loaded in the software Scaffold (Version 4.8.8, Proteome Software Inc., Portland, OR). To ensure significant protein identification the protein probability filter was set to protein FDR: 5% peptide FDR:1% decoy. Protein probabilities were assigned by the Protein Prophet algorithm [43]. Proteins that contained similar peptides and could not be differentiated based on MS/MS analysis alone were grouped to satisfy the principles of parsimony. Raw data from MEEC/MESC immunoprecipitations were analyzed by Peaks Studio 10.6. Therefore, spectra were searched against a Swiss Prot mouse database (version 2024, including 21708 entries). MS^1^ and MS^2^ spectra were matched with a mass tolerance of 10 ppm for precursor masses and 0.7 Da for peptide fragment ions. Tryptic digest and up to three missed cleavage sites were allowed, carbamidomethylation on cysteine were used as a fixed modification for database search and deamidation of asparagine and glutamine, acetylation of lysine and oxidation of methionine were used as variable modifications.

### Ca^2+^ imaging in isolated MEECs

MEECs from *Trpv6*^*−/−*^, *Trpv6*^*mt/mt*^ and wild-type mice, plated on 25 mm collagen-coated coverslips, were loaded with 5 µM Fura-2 AM (Invitrogen) for 30 min at RT in Ringer’s solution containing 115 mM NaCl, 5 mM KCl, 2 mM CaCl_2_, 2 mM MgCl_2_, 10 mM HEPES, 10 mM glucose, pH 7.4. Subsequently, the coverslips were placed in a bath chamber, washed three times with Ringer´s solution and mounted with a volume of 300 µL of nominal Ca^2+^-free or Ca^2+^-containing Ringer´s solution (see start of experiments in Fig. 4, with or without ORAI channel blocker GSK7975A or BTP2 (both Merck)) on the stage of a Zeiss AxioVert S100 inverted microscope equipped with a Fluar-20x/0.75 objective (Zeiss), a monochromator (Polychrom V, TILL Photonics) and a charge-coupled device camera (Clara CCD, Andor Technology). Changes in intracellular Ca^2+^ concentration were recorded at 1 Hz as fluorescence (> 440 nm) ratio (F340/F380), calculated during 50 ms excitation at 340 and 380 nm after subtraction of the background. Individual cells were selected as regions of interest (ROI) with Live Acquisition (LA) software (TILL Photonics) and F340/F380 was plotted versus time. The SERCA inhibitor cyclopiazonic acid (CPA) and CaCl_2_ were added to the bath to reach final concentrations as indicated. For analysis, peak amplitude and area under the curve of Ca^2+^ release and Ca^2+^ influx were calculated after subtraction of the baseline right before store depletion (CPA) and Ca^2+^ re-addition as DF340/F380 and DF340/F380 x s, respectively.

### HEK-293 and COS-7 cell culture and transfection

HEK-293 cells (ATCC, CRL 1573) and COS-7 cells (ATCC, CRL-1651), obtained from the American Type Culture Collection (ATCC, Manassas, VA), were cultured in 75 cm^2^ flasks in minimal essential medium (MEM HEK-293) and Dulbecco’s modified eagles medium (DMEM COS-7) (both Life Technologies, Carlsbad, USA) containing 10% fetal calf serum (FCS; Life Technologies) at 37 °C and 5% CO_2_. For transient transfection, cells were grown in 3 cm diameter culture dishes until 80% confluence and then transiently transfected with 4 µg of pCAGGS-mTRPV6-IRES-GFP cDNA in 5 µL of the PolyFect^®^ reagent (Qiagen, Hilden, Germany). Dishes were trypsinized and transfected cells were plated on 10 mm PLL-coated glass coverslips for patch clamp experiments, performed on GFP-expressing cells 48 h after transfection. For western blot analysis 24 h after transfection cells were resuspended with 150 µL denaturing sample buffer (final concentration: 60 mM Tris HCl, pH 6.8, 4% SDS, 10% glycerol including 0.72 M β-mercaptoethanol) and incubated at 60 °C for 20 min before applying on SDS-PAGE [23].

### Electrophysiological recordings

Whole cell currents of MEECs and HEK-293 cells were recorded in the tight seal patch clamp configuration using an EPC-9 amplifier (HEKA Electronics, Lambrecht, Germany). Patch pipettes were pulled from glass capillaries GB150T-8P (Science Products, Hofheim, Germany) at a PC-10 micropipette puller (Narishige, Tokyo, Japan) and had resistances between 3 and 5 MΩ when filled with internal solution (in mM): 120 Cs-Glutamate, 8 NaCl, 1 MgCl_2_, 10 HEPES, 10 Cs-BAPTA, pH adjusted to 7.2 with CsOH. Standard external solution contained (in mM): 140 NaCl, 10 CaCl_2_, 10 CsCl, 2 MgCl_2_, 10 HEPES, 10 glucose, pH adjusted to 7.2 with NaOH. Where indicated divalent-free (DVF) saline, based on standard external solution without Ca^2+^ and Mg^2+^ but with 10 mM EGTA, was pressure applied directly onto the patch clamped cell by a patch pipette with a slightly broken tip. Osmolarity of all solutions ranged between 285 and 305 mOsm. Voltage ramps of 50 ms duration spanning a voltage range from −100 to +100 mV were applied at 0.5 Hz from a holding potential (V_h_) of 0 mV over a period of up to 360 s using the PatchMaster 2.90 software (HEKA, Reutlingen, Germany). All voltages were corrected for a 10 mV liquid junction potential. Currents were filter at 2.9 kHz and digitized at 100 µs intervals. Capacitive currents and series resistance were determined and corrected before each voltage ramp using the automatic capacitance compensation of the EPC-9. Inward and outward currents were extracted from each individual ramp current recording by measuring the current amplitudes at −80 and +80 mV, respectively, and plotted versus time. Representative current-voltage relationships (IVs) were extracted at indicated time points. To obtain voltage relationships of net currents developing during application of DVF saline and 20 s after its removal (Fig. 6b and e), currents before DVF condition were subtracted. Net current voltage relationships of mTRPV6 currents in HEK-293 cells (Fig. 6d) were obtained by subtracting the basic current after break-in. All currents were normalized to the cell size (pA/pF).

### Confocal Ca^2+^ imaging on isolated uteri *in situ*

Uterine horns of adult female *Trpv6*-IC/eR26-GCaMP3 and *Trpv6*^*−/−*^-IC/eR26-GCaMP3 mice in the estrus phase of the cycle were dissected and placed in an ice-cold Ringer’s solution containing 115 mM NaCl, 5 mM KCl, 2 mM MgCl_2_, 2 mM CaCl_2_, 10 mM HEPES, 10 mM glucose, pH 7.4. Residual connective and adipose tissue were removed, and the uteri were dissected and opened longitudinally to expose the endometrium. The epithelial layer of the endometrium was intactly scraped off with a scalpel blade to detach it from the smooth muscle layer of the uterus. Cytosolic Ca^2+^ imaging experiments were performed using an upright confocal microscope (Zeiss, LSM 710) equipped with a multi-line argon laser and a water immersion 20x objective (Zeiss, Plan-Apochromat). Ca^2+^-dependent GCaMP3 fluorescence (493–598 nm, excitation 488 nm), appearing exclusively in MEECs, was recorded at 2 Hz at 2 mM and 0.5 mM extracellular Ca^2+^ (see start of experiments in Fig. 5). Ca^2+^-free solution containing 0.5 mM EGTA and 10 µM CPA were applied as indicated. All solutions were gravity applied through a valve controller system (Warner Instruments, VC-6 Valve Controller and TTL switch, CED Micro1401-3). Regions of interest were marked with the ZEN Black software (Zeiss) and the fluorescence recordings were analyzed with ImageJ and plotted as F/F_0_ (fluorescence intensity F divided by the basic fluorescence at the beginning of the experiment F_0_) versus time.

### Statistics

Ca^2+^ imaging data were analyzed using OriginPro 2021b (OriginLab Corporation, Northampton, MA, USA) and Igor Pro 6.31 (WaveMetrics, Portland, OR, USA). Fitmaster 2.90 software (HEKA, Reutlingen, Germany) and GraphPad Prism 9 (GraphPad software, Boston, MA, USA) were used to analyze and plot patch clamp data, as well as for statistical testing and graphing.

Current traces vs. time are shown as means ± standard error of the mean (SEM). The normality of data distribution was tested using the Shapiro-Wilk test. Data of normal distribution (parametric) were plotted as bar graphs showing means ± standard deviation (SD) and the significance was assessed either by unpaired two-tailed Student’s t-test (two groups) or one-way ANOVA with Tukey’s multiple comparison test (more than two groups). Non-parametric data are shown as Tukey’s box and whiskers with median and boxes, which extend from the 25th to the 75th percentile (interquartile range [IQR]). Whiskers are extended to the most extreme data point that is no more than 1.5 x IQR from the edge of the box, and outliers beyond the whiskers are depicted as dots. The significance of the non-parametric data was evaluated by Mann-Whitney U tests (two groups) or Kruskal Wallis with Dunn’s multiple comparison tests (more than two groups). *P* values of less than 0.05 were considered statistically significant. Final figures were prepared with CorelDraw (Alludo, Ottawa, ON, Canada).

## Results

### TRPV6-deficiency impairs fecundity in female mice

**Fig. 1:**
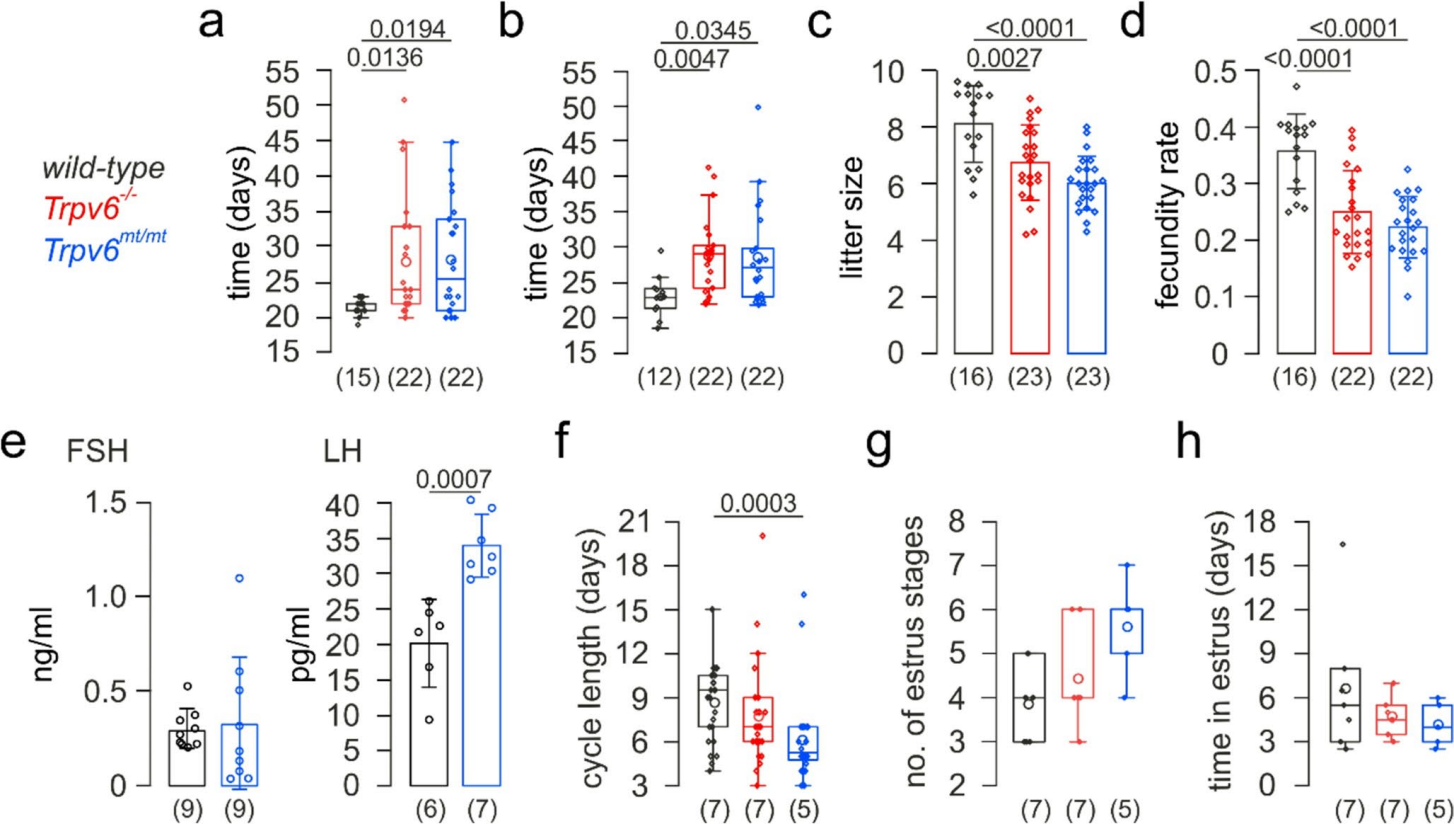
Trpv6-deficiency impairs fecundity in female mice. Waiting time until first pregnancy (**a**), interval between litters (**b**), litter size (**c**), fecundity rate (**d**), blood concentration of FSH (follicle-stimulating hormone) and LH (luteinizing hormone; **e**), estrous cycle length (**f**), number (no.) of estrus stages (**g**) and time spent in estrus during a period of 35 days (**h**) in female *wild-type*, *Trpv6*^*−/−*^ and *Trpv6*^*mt/mt*^ mice. Non-parametric and parametric data are presented as Tukey box (interquartile range (IQR) from 25th to the 75th percentile) and whiskers (extended to the most extreme data point no more than 1.5 x IQR from edge of the box) with median (a, b, f-h) and bar graphs with mean ± SD (c-e), respectively, and are statistically analyzed by Kruskal-Wallis (a, b, f-h), one-way ANOVA (c, d) and Student´s t-test (e). Numbers on top of the graphs represent the P values. Dots and circles in a, b and f-h represent single and mean values, respectively. Circles in d and e represent single values. The numbers of analyzed matings (a-c) and individual mice (d-h) are indicated below the boxes and bars

As previously shown [3], TRPV6 is expressed in the decidua during pregnancy. The decidua is the essential prerequisite for perception and maintenance of the embryo. Homozygous *Trpv6*^*−/−*^-deficient female mice or female mice homozygously carrying a single-point mutation within the channel pore of TRPV6 (D581A), leading to a non-functional TRPV6 channel (*Trpv6*^*mt/mt*^), conceived their very first litter after the first mating in average about 5 days later as compared to the respective wild-type mice (Fig. 1a, open circles). In addition, the time between consecutive litters was increased by more than 20% and the average litter size was decreased from 8.1 pups in wild-type to 6.7 and 6.0 pups in *Trpv6*^*−/−*^ and *Trpv6*^*mt/mt*^ mice, respectively (Fig. 1b, c). Calculated from these parameters (see methods) the fecundity rate was significantly reduced in mice lacking functional TRPV6 channels (Fig. 1d).

### In estrus, TRPV6 is present in MEECs

While in *Trpv6*^*mt/mt*^ mice the blood level for LH but not for FSH was significantly increased (Fig. 1e) and the estrus cycle length decreased (Fig. 1f), neither the number of estrus stages (Fig. 1g) nor the time in estrus (Fig. 1h; Fig. S1) was different between the two functional TRPV6-KO models (*Trpv6*^*−/−*^ and *Trpv6*^*mt/mt*^) and wild-type mice.

**Fig. 2:**
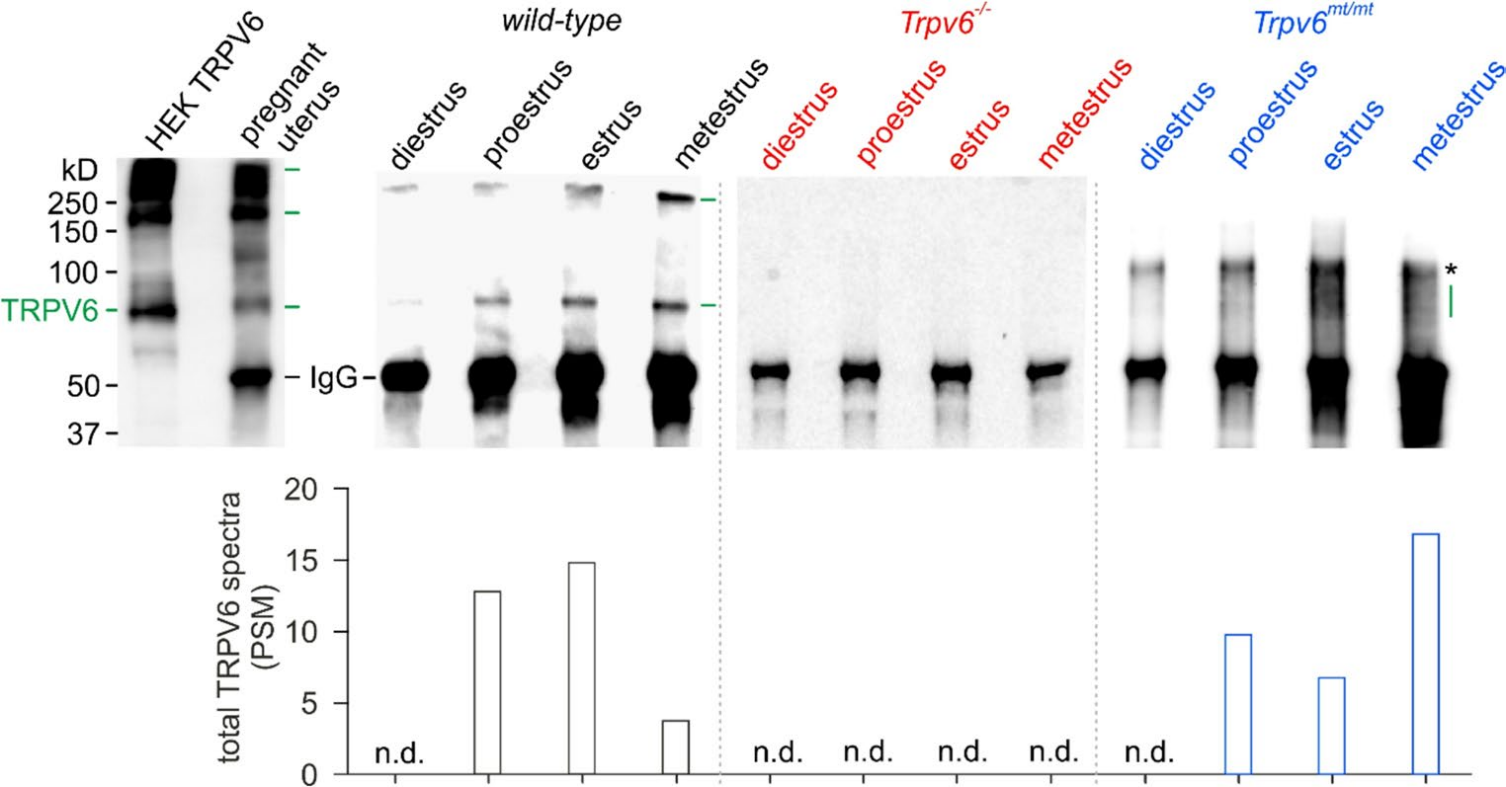
Estrous cycle-dependent expression of TRPV6 in the mouse uterus. Upper panel: Western blot detection of TRPV6 protein in lysates of HEK-293 cells transfected with mTRPV6 cDNA or in elutes after immunoprecipitation (IP) of TRPV6 from pregnant uteri (E12.5) and during all four stages of the estrous cycle from *wild-type*, *Trpv6*^*−/−*^ and *Trpv6*^*mt/mt*^ uteri. For IP an antibody directed against the N-terminus and for detection an antibody against the C-terminus of TRPV6 was used. The green lines indicate mono- and multimers of TRPV6 proteins (also seen as smeared bands in elutes from *Trpv6*^*mt/mt*^ uteri), while the asterisk indicates IgG multimers. Lower panel: total number of tryptic TRPV6 peptide spectra (PSM) detected by mass spectrometry after TRPV6-specific IP from murine uteri (*n* = 2). Note that TRPV6-specific tryptic peptides are detected in *wild-type* and *Trpv6*^*mt/mt*^ (pore mutant) but not in *Trpv6*^*−/−*^ (global knockout) uteri (n.d. = no detection)

**Fig. 3:**
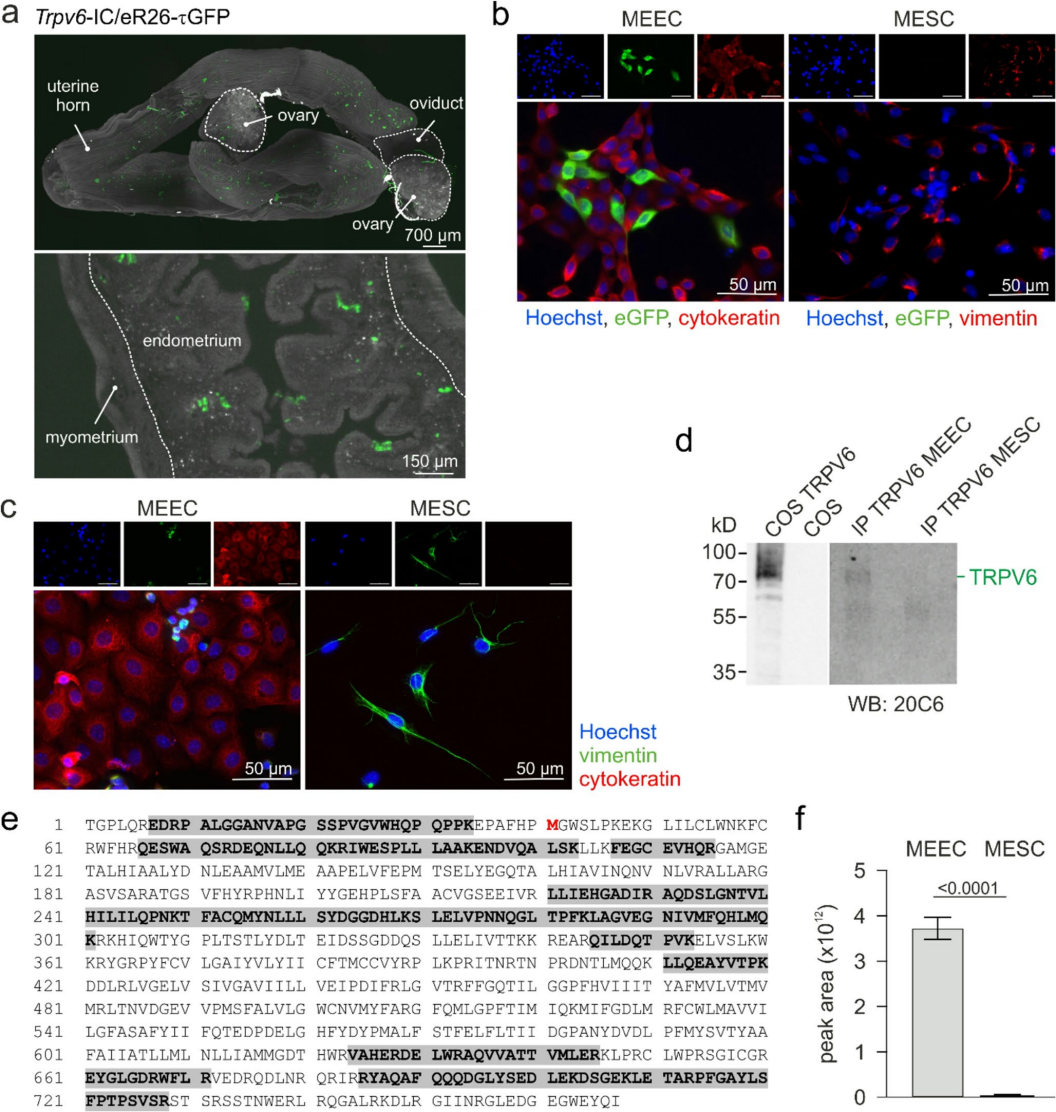
TRPV6 is expressed in MEECs, but not in MESCs. (**a**) iDISCO cleared uterus of a *Trpv6*-IC/eR26-τGFP mouse. 2D projection of a 3D image stack from uterus with ovaries (top) and 2D image of the endometrium at higher magnification (bottom). GFP-expressing cells (green) are mainly observed in the epithelial cell layer of the endometrium. (**b**) Immunostaining of eGFP (green) in endometrial epithelial (MEEC) and stromal (MESC) cells, identified by positive staining against cytokeratin and vimentin (red), cultured from uteri of *Trpv6*-IC/eR26-τGFP mice. (**c**) Staining against cytokeratin (red) and vimentin (green) in MEEC and MESC cultures, isolated from wild-type mice uteri. In b and c Hoechst was used to stain nuclei (blue; top: single pictures; bottom: merged pictures). (**d**) Western blot of lysates from TRPV6 cDNA-transfected and non-transfected COS-7 (COS) cells and elutes from MEEC and MESC cultures after immunoprecipitation (IP) with TRPV6 antibodies (N-terminal) incubated with the monoclonal C-terminal TRPV6 antibody (20C6). (**e**) Protein sequence of mouse TRPV6 (SwissProt: Q91WD2) with amino acids identified by MS/MS fragmentation in MEECs highlighted (bold letters, grey), which cover 33% of the sequence (3 technical replicates). Notably, one peptide detected by mass spectrometry is located upstream of the initially annotated initiation methionine [22], which is shown in red. (**f**) Comparison of the semiquantitative TRPV6 protein abundance detected by MS^2^ analysis in the elutes after immunoprecipitation from MEECs and MESCs. The protein abundance is shown as sum of all detected parent TRPV6 peptide areas (*n* = 3 unpaired t-test, bar graphs with mean + SD)

We recently identified TRPV6 in a subset of endometrial cells in the uterus of pregnant mice ([21], see also Fig. 3a). Western Blot and mass spectrometry analysis of TRPV6 antibody-enriched protein lysates of isolated uteri from wild-type and *Trpv6*^*mt/mt*^ mice revealed the presence of TRPV6 protein at proestrus, estrus and metestrus but not at diestrus, demonstrating a cycle-dependent expression of TRPV6 (Fig. 2). The MS results also show that TRPV6 abundance in the uterus of wild-type and *Trpv6*^*mt/mt*^ mice is comparable, as the number of TRPV6 peptides identified across all cycle stages is similar (see Fig. 2, lower panel). Uteri from *Trpv6*^*−/−*^ mice lack the TRPV6 protein. Since protein expression in wild-type mice was high at estrus, we focused on a possible functional expression of TRPV6 in the uterus of mice at that cycle stage.

To substantiate the presence of TRPV6 in the uterus, we isolated uteri from adult female *Trpv6*-IC/eR26-τGFP mice at estrus and visualized GFP-positive cells in the endometrial cell layers using immunolabeling-enabled three-dimensional imaging of solvent-cleared organs (iDISCO [44]), (Fig. 3a). The mouse endometrium consists of two major cell types, epithelial and stromal cells. TRPV6-positive cells apparently appeared in the epithelial cell layer. To identify the TRPV6-expressing cells, we isolated the uteri of adult female *Trpv6*-IC/eR26-τGFP mice at estrus and cultured the mouse endometrial epithelial cells (MEECs) and stromal cells (MESCs), each separately. MEECs and MESCs were identified by a positive staining of cytokeratin and vimentin, respectively (Fig. 3b). While the MESC culture was devoid of GFP-positive cells, about 10% of the MEECs were GFP-positive, i.e. the TRPV6 promotor had been active. For further experiments we prepared MEEC and MESC cultures from wild-type, *Trpv6*^*−/−*^ and *Trpv6*^*mt/mt*^ mice. Figure 3c and Supplementary Fig. 2 demonstrate highly enriched MEEC and MESC cultures, verified by positive staining against cytokeratin and vimentin, which did not differ between the three genotypes (Fig. 3c and Fig. S2). Western blot and mass spectrometric analysis of TRPV6 immunoprecipitations from wild-type cells show a > 100-fold higher expression of TRPV6 in MEECs compared to MESCs (Fig. 3d-f). In the later only few and low abundant TRPV6 peptide spectra were identified, which could be due to low contamination with MEECs. Notably, one peptide detected by mass spectrometry is located upstream of the initially published starting methionine (Fig. 3e, red). For positive and negative control of the Western blot, TRPV6-cDNA-transfected and non-transfected COS cells were used (Fig. 3d).

### TRPV6 contributes to Ca^2+^ influx in MEECs

**Fig. 4:**
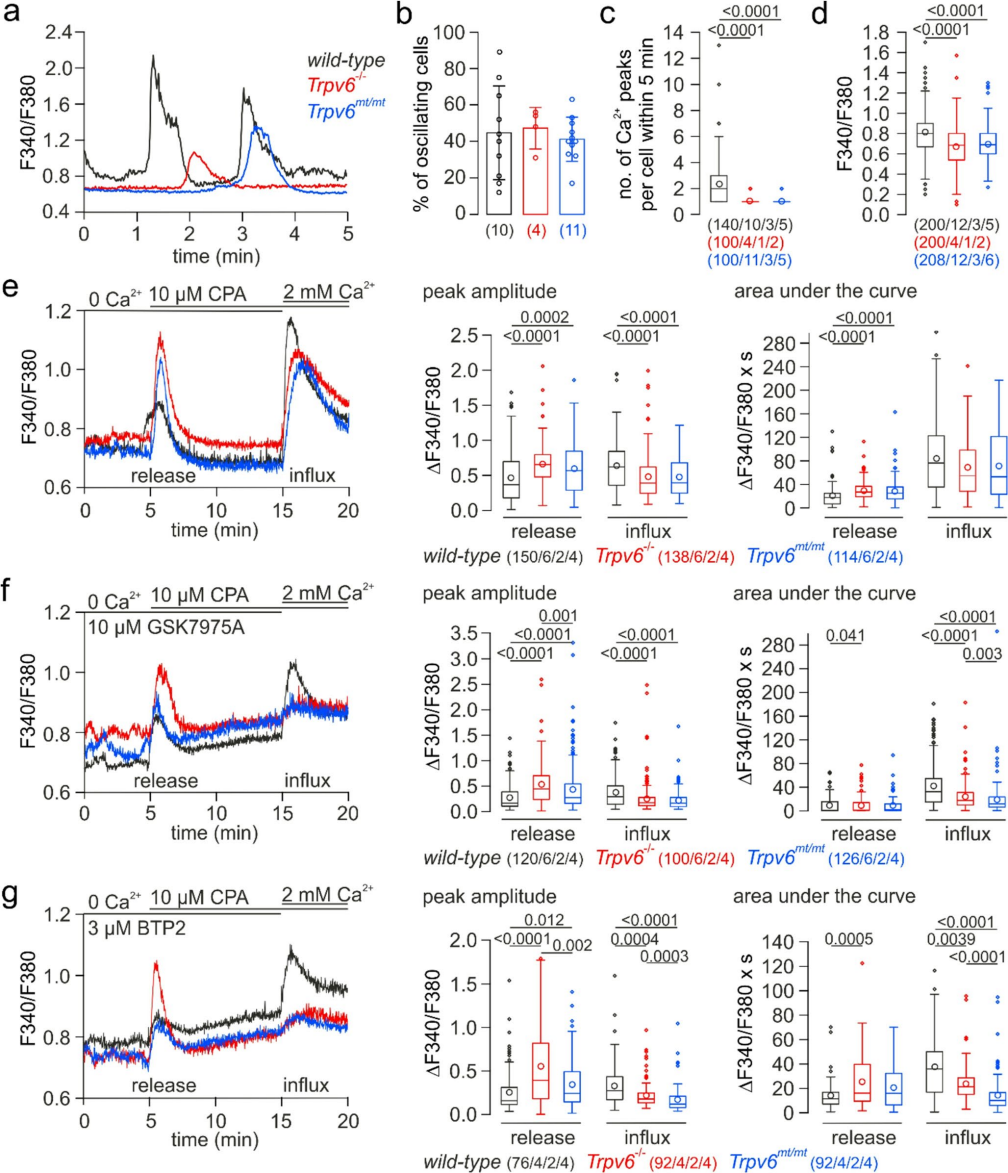
TRPV6 contributes to Ca^2+^ influx in cultured MEECs. Representative cytosolic Ca^2+^ recordings (F340/F380) from MEECs isolated from *wild-type* (black), *Trpv6*^*−/−*^ (red) and *Trpv6*^*mt/mt*^ (blue) mice in the presence of 2 mM extracellular Ca^2+^ (**a**) and in the nominal absence of extracellular Ca^2+^ (0 Ca^2+^) with subsequent store-depletion by 10 µM cyclopiazonic acid (CPA) and 2 mM Ca^2+^ re-addition (**e**-**g**, left panels) in the absence (e) and presence (f, g) of ORAI inhibitors GSK7975A (10 µM, f) and BTP2 (3 µM, g). (**b**-**d**) Percentage of oscillating cells (b), number (no.) of cytosolic Ca^2+^ peaks per active cell within 5 min (c) and basic Ca^2+^ levels (d) analysed from experiments as shown in a. (**e**-**g**, right panels) Peak amplitude and area under the curve of the baseline-subtracted Ca^2+^ signals (ΔF340/F380) after CPA-induced store depletion (release) and Ca^2+^ re-addition (influx) analysed from experiments as shown in the respective left panels. Non-parametric and parametric data are presented as Tukey box (interquartile range (IQR) from 25th to the 75th percentile) and whiskers (extended to the most extreme data point no more than 1.5 x IQR from edge of the box) with median (c-g) and bar graphs with mean ± SD (b), respectively, and are statistically analyzed by Kruskal-Wallis (c-g) and one-way ANOVA tests (b). Dots in b represent single values and dots and circles in c-g represent single values beyond the whiskers and mean values, respectively. Numbers on top of the graphs represent the P values. The numbers in b represent the number of measured dishes, and in c-f (n/x/y/z) the number of analyzed cells (n), recorded in (w) dishes from (y) independent cultures, prepared from (z) mice

To prove a possible contribution of TRPV6 in cytosolic Ca^2+^ signaling in the mouse endometrial epithelial cells, we isolated MEECs from adult wild-type, *Trpv6*^*−/−*^ and *Trpv6*^*mt/mt*^ female mice at estrus and performed Ca^2+^ imaging experiments using the Ca^2+^-sensitive fluorophore Fura-2. No apparent morphological alterations were observed in the isolated uteri of the three genotypes. About 45% of the cultured MEECs revealed spontaneous Ca^2+^ signals (Fig. 4a, b; traces in a represent Ca^2+^ signals from single cells). While the percentage of spontaneously active MEECs did not differ between the genotypes, MEECs from wild-type mice revealed in average two Ca^2+^ peaks and MEECs from *Trpv6*^*−/−*^ and *Trpv6*^*mt/mt*^ mice only one Ca^2+^ peak within 5 min (Fig. 4b, c). In addition, the basic Ca^2+^ level in wild-type cells was slightly but significantly higher than in *Trpv6*^*−/−*^ and *Trpv6*^*mt/mt*^ cells (Fig. 4d). To prove whether TRPV6 contributes to Ca^2+^ influx in MEECs, we depleted the intracellular Ca^2+^ stores by the inhibition of Ca^2+^ re-uptake into the stores using the SERCA inhibitor cyclopiazonic acid (CPA) in the absence of extracellular Ca^2+^ and subsequently re-added Ca^2+^. In *Trpv6*^*−/−*^ and *Trpv6*^*mt/mt*^ MEECs the peak amplitude of the Ca^2+^ influx after Ca^2+^ re-addition was significantly, and its area under the curve tendentially reduced as compared to wild-type cells (Fig. 4e; traces represent Ca^2+^ signals from single cells).

Ca^2+^ store depletion activates store-operated Ca^2+^ channels, especially ORAI1-3 [45], which significantly contribute to the Ca^2+^ influx after Ca^2+^ re-addition. While in the presence of GSK7975A and BTP2 (YM-58483), two well established inhibitors of store-operated Ca^2+^ channels [46, 47], Ca^2+^ entry upon Ca^2+^ re-addition after store depletion in *Trpv6*^*−/−*^ and *Trpv6*^*mt/mt*^ cells almost completely disappeared, wild-type MEECs still revealed a significant Ca^2+^ influx (Fig. 4f, g; traces represent Ca^2+^ signals from single cells). The data suggests that TRPV6 essentially contributes to Ca^2+^ influx in wild-type MEECs. However, the CPA-mediated Ca^2+^ release in the absence and presence of GSK7975A and BTP2 was increased in *Trpv6*^*−/−*^ and *Trpv6*^*mt/mt*^ MEECs as compared to the wild-type cells (Fig. 4e-g). Apparently, the functional ablation of TRPV6 leads to a higher Ca^2+^ content of the CPA-sensitive intracellular Ca^2+^ stores.

### TRPV6 contributes to spontaneous Ca^2+^ oscillations in MEECs *in situ*

**Fig. 5:**
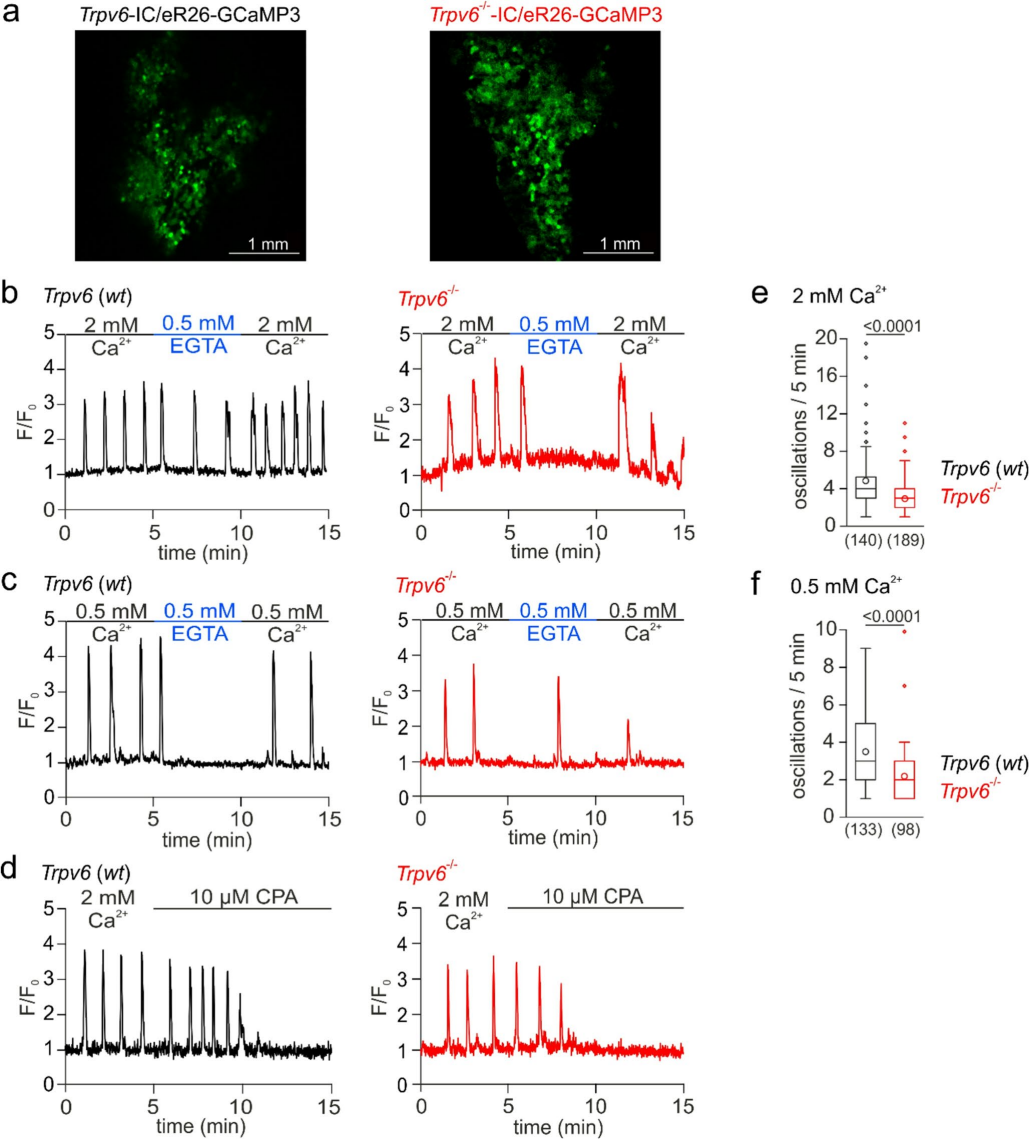
***Trpv6***-deficiency lowers frequency of Ca^2+^ oscillations in MEECs *in situ*. (**a**) GCaMP3 fluorescence in isolated uteri from *Trpv6*-IC/eR26-GCaMP and *Trpv6*^*−/−*^-IC/eR26-GCaMP mice, i.e. in cells where the TRPV6 promotor had been active. (**b-d**) Representative cytosolic Ca^2+^ changes (F/F_0_) detected in GCaMP3-expressing cells from uteri of *Trpv6*- (*Trpv6* (*wt*), black) and *Trpv6*^*−/−*^-IC/eR26-GCaMP3 mice (*Trpv6*^*−/−*^, red) at 2 mM (b, d) and 0.5 mM (c) extracellular Ca^2+^. At the indicated time points Ca^2+^-free saline with 0.5 mM EGTA (b, c; blue) or 10 µM cyclopiazonic acid (CPA; d) were applied. (**e**, **f**) The box (interquartile range (IQR) from 25th to the 75th percentile) and whiskers (extended to the most extreme data point no more than 1.5 x IQR from edge of the box) blots with median depict the number of distinct Ca^2+^ peaks (oscillations) within 5 min in 2 mM (e) and 0.5 mM (f) external Ca^2+^. The data was statistically analyzed by Mann-Whitney’s tests. P values are shown on top of the plots. Dots and circles in e and f represent single values beyond the whiskers and mean values, respectively. Measurements were obtained from 7 preparations (uteri), i.e. 7 animals per genotype for each experimental condition and include the number of cells as shown in brackets

To study cytosolic Ca^2+^ signaling in TRPV6-expressing and TRPV6 knock-out cells in the endometrium in situ, we prepared uteri from adult female *Trpv6*-IC/eR26-GCaMP3 and *Trpv6*^*−/−*^-IC/eR26-GCaMP3 mice at estrus, which express the endogenous Ca^2+^-indicator GCaMP3 in a Cre-dependent manner in *Trpv6*^*+/+*^-Cre and *Trpv6*^*−/−*^-Cre cells, i.e. in the MEECs (Fig. 5a). For Ca^2+^ imaging, the uterus tube was cut open and spread flat into the bath chamber with the endometrial layer on top. 30–50% of the cells in the endometrial layer of the uteri prepared from the *Trpv6*- and *Trpv6*^*−/−*^-IC/eR26-GCaMP3 mice expressed the fluorescent Ca^2+^ indicator. The uteri of both genotypes revealed regular cytosolic Ca^2+^ oscillations in the GCaMP3-positive MEECs, which disappeared over time in the absence of extracellular Ca^2+^ (Fig. 5b, c) and after depletion of the intracellular Ca^2+^ stores by CPA (Fig. 5d; all traces represent Ca^2+^ signals from single cells). While the reduction of extracellular Ca^2+^ generally resulted in less spontaneous Ca^2+^ signals in the MEECs of both genotypes, *Trpv6*-deficient cells revealed significantly reduced frequencies of basic Ca^2+^ oscillations as compared to *Trpv6* wild-type cells in both 2 mM and 0.5 mM extracellular Ca^2+^ (Fig. 5e, f). The data suggest that both Ca^2+^ release and Ca^2+^ influx contribute to the spontaneous Ca^2+^ oscillations in MEECs in situ, and that TRPV6 channel activity is significantly involved in this process.

### TRPV6-dependent whole-cell currents in wild-type MEECs

**Fig. 6:**
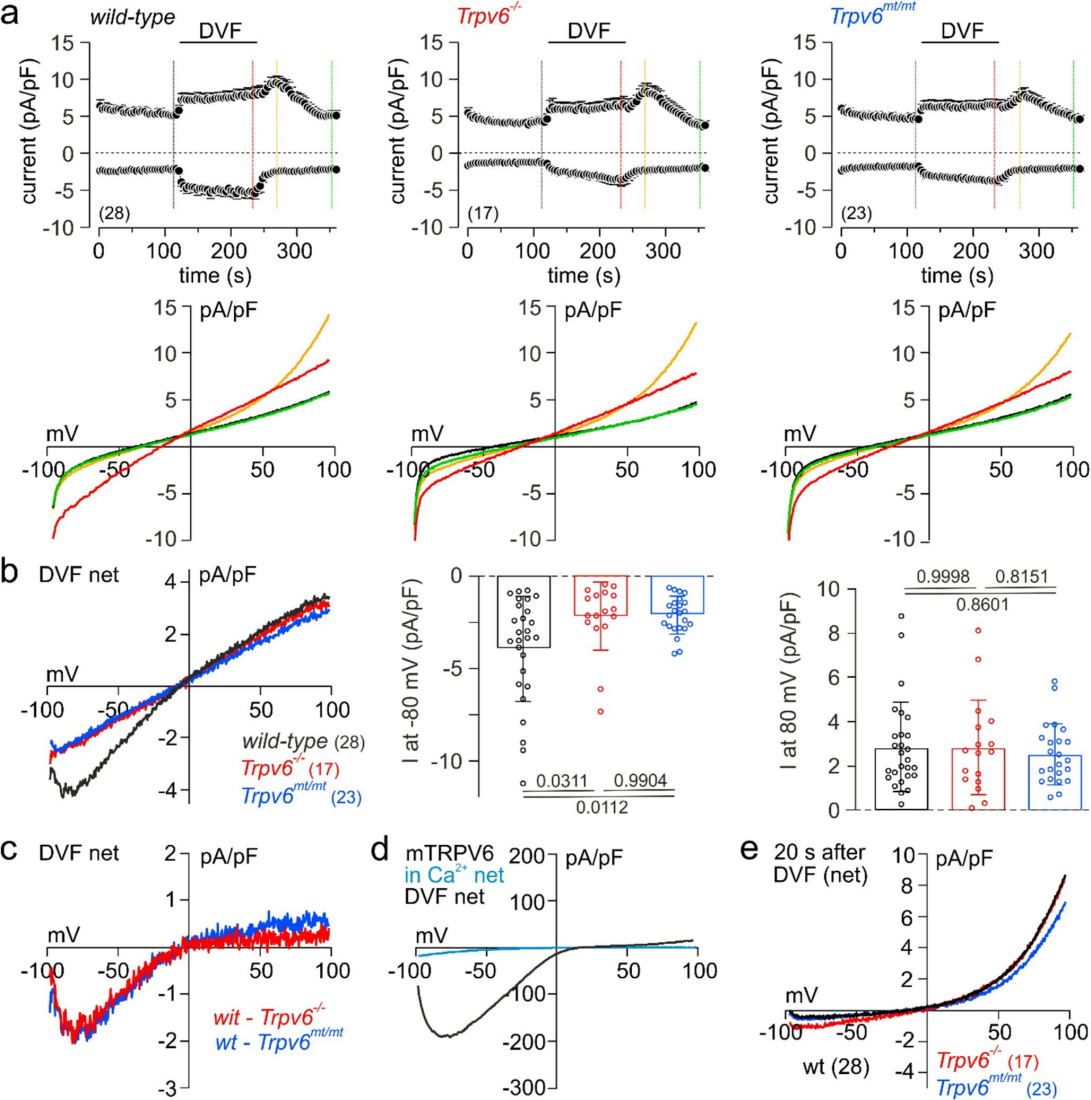
MEECs reveal a TRPV6-dependent whole-cell current at DVF conditions. (**a**) In- and outward currents recorded at −80 and 80 mV during voltage ramps spanning from − 100 to 100 mV, applied at 0.5 Hz, and plotted versus time in MEECs isolated from *wild-type* (*wt*), *Trpv6*^*−/−*^ and *Trpv6*^*mt/mt*^ mice (top panels). Bars indicate the application of divalent-free saline (DVF). Current-voltage-relationships (IVs) at the time points as indicated by the colored lines are shown in the lower panels. Black traces are behind the green traces. (**b**) IVs (left) and amplitudes at −80 mV (middle) and 80 mV (right) of the net DVF-mediated currents (currents just before DVF had been subtracted). (**c**) IV of the net current appearing in DVF saline only in *wild-type* but not in *Trpv6*^*−/−*^ and *Trpv6*^*mt/mt*^ MEECs (calculated from traces in b: *wt* minus *Trpv6*^*−/−*^ (red) and *wt* minus *Trpv6*^*mt/mt*^ (blue)). (**d**) IVs of the net whole-cell currents before (in Ca^2+^, light blue) and in DVF saline (black) in HEK-293 cells transfected with mTRPV6 cDNA. (**e**) IVs of the net current 20 s after DVF in *wt*, *Trpv6*^*−/−*^ and *Trpv6*^*mt/mt*^ MEECs. All currents were normalized to the cell size (pA/pF). Data show means ± SEM (a, top), means ± SD (bar graphs in b, circles represent single values) or just means (all IVs). The parametric data in b were analyzed by one-way ANOVA tests, with P values below or on top of the bars. The numbers of analyzed cells is shown in brackets

To the best of our knowledge, no distinct endogenous TRPV6-dependent whole-cell Ca^2+^ current has yet been recorded in any primary cell type, i.e. cells acutely prepared from living tissue expressing *Trpv6*, controlled by the corresponding cell type from *Trpv6* knockouts. We recorded whole-cell currents in MEECs isolated from wild-type, *Trpv6*^*−/−*^ and *Trpv6*^*mt/mt*^ mice, but a TRPV6-like Ca^2+^ current was not detectable either (Fig. 6a). It has been shown, that in the absence of extracellular divalent cations the Ca^2+^-selective pore of TRPV6 becomes permeable to monovalent cations, resulting in a significant boost of the inward current [22, 23, 48]. Upon removal of divalent cations, i.e. during the application of divalent-free saline (DVF), a significant whole-cell current appeared in the MEECs of all three genotypes (Fig. 6a, top panel; current voltage relationships at different time points are shown in the lower panel). However, the net DVF-mediated inward current (but not the outward current) in wild-type MEECs was significantly larger than in *Trpv6*^*−/−*^ and *Trpv6*^*mt/mt*^ cells (Fig. 6b). While this (net) extra current in the presence of functional TRPV6 ion channels is very small (in average less than 2 pA/pF), its current-voltage relationship (Fig. 6c, DVF-mediated net current in wild-type minus *Trpv6*^*−/−*^ (red) or minus *Trpv6*^*mt/mt*^ (blue)) reveals exactly the same characteristic course as at divalent-free conditions for human TRPV6 transiently expressed in HEK-293 cells (Fig. 6d). The other part of the DVF-mediated current and the transient, mainly outwardly directed current after re-addition of divalent-containing bath solution reveal similar shapes and amplitudes in all three genotypes (Fig. 6a, b and e) and apparently do not depend on TRPV6.

## Discussion

The TRPV6 ion channel is crucial for male mouse fertility [1, 2] and, in pregnant female mice, for a sufficient supply of Ca^2+^ to the embryo, which is important for fetal growth, bone calcification and bone development [3]. In addition, TRPV6 controls extracellular matrix structure of the placental labyrinth [49]. The present study shows that mice lacking functional TRPV6 channels have problems with their first pregnancy, have a longer latency between pregnancies and produce fewer offspring, a phenomenon known as impaired fecundity (Fig. 1).

Embryo transfer experiments revealed that TRPV6 in both the fetal tissue (labyrinth trophoblasts, yok sac) and the maternal tissue (decidua) of the placenta contributes to murine embryonic development and bone mineralization [3]. In that previous study, we investigated the function of TRPV6 in labyrinth trophoblasts, i.e. in the fetal part of the placenta. Here we show the functional expression of TRPV6 in the maternal tissue, namely in epithelial cells of the endometrium, the inner layer of the uterus.

TRPV6 has already been identified in pig luminal endometrial cells and bovine endometrium during pregnancy [50, 51] and in sheep endometrial tissue during the proliferative phase [52]. A cycle-dependent expression of TRPV6 has been shown for mouse, rat and human endometrium with the highest mRNA level at proestrus and estrus in mouse [53], diestrus in rat [54] and during the proliferative phase in human endometrial tissue [55]. We identified TRPV6-positive cells in the decidua of pregnant *Trpv6*-IC/eR26-tGFP mice [3] and the endometrial layer of uteri in *Trpv6*-IC/eR26-tGFP and *Trpv6*-IC/eR26-GCaMP3 mice at estrus (Figs. 3a and 5a). In addition, and in agreement with De Clercq et al. [53], we found the highest levels for the TRPV6 protein at proestrus and estrus (Fig. 2). The expression studies suggest that TRPV6 is especially needed for proper endometrial function during the proliferative phase of the estrous cycle and during pregnancy.

The mouse endometrium consists of epithelial cells (MEECs) and stromal cells (MESCs). The present study revealed a significant expression of TRPV6 proteins mainly in MEECs (Fig. 3). Again, this is in good agreement with De Clercq et al. who reported on the distinct mRNA expression of TRPV4, TRPV6 and TRPM6 in mouse and human endometrial epithelial cells, while endometrial stromal cells mainly express TRPV2, TRPC1/4 and TRPC6 mRNA [53].

During the estrous cycle, the human endometrium transforms into the decidua, which enables the implantation of the blastocyst and a successful pregnancy. In mice, decidualization has been shown to depend on the presence of a blastocyst in the uterine lumen [30–32]. Thus, the blastocyst must send a signal to the MEECs, which further signal to the underlying stromal cells, to finally trigger and maintain decidualization. Pre-implantation blastocysts are shown to secrete diverse hormones, growth factors and proteases which might serve as signaling molecules in this process [56–59]. As part of the sensing and signaling pathway in the MEECs Ca^2+^ influx had been suggested, initiated by activation of the epithelial sodium channel (ENaC) and mediated either by a depolarization-dependent activation of voltage-gated Ca^2+^ channels (VGCC) or a reverse mode of the sodium calcium exchanger (NCX) [60, 61]. Cytosolic Ca^2+^ signaling in the MEECs has already been shown to be important for the adhesion between the embryonic trophoblasts and the endometrial epithelial cells [29, 62]. However, Hennes et al. did not find any evidence for the involvement of ENaC, VGCC and NCX to Ca^2+^ influx in MEECs [63]. Instead, they identified the protease-activated receptor 2 (PAR-2) as the molecular entity initiating cytosolic Ca^2+^ oscillations in MEECs which depend on the phospholipase C (PLC)/inositol-1,4,5-trisphospate receptor (IP_3_R)/store-operated Ca^2+^ entry (SOCE) pathway upon secretion of proteases from the invading blastocyst. In their experiments, Ca^2+^ oscillations in primary MEECs had been induced by trypsin. SOCE and Orai1 expression had also been shown in Ishikawa cells, a human endometrial adenocarcinoma cell line with characteristics of luminal endometrial epithelial cells [64]. In addition, Piezo1, a mechanosensitive Ca^2+^ permeable cation channel, functionally identified in mice and human endometrial epithelial cells, has also been suggested as potential signaling transducer between the blastocyst and the endometrium [65].

However, here we identified for the first time functional TRPV6 channels and their contribution to Ca^2+^ influx and spontaneous Ca^2+^ signaling in living MEECs in vitro and in situ (Figs. 4, 5 and 6). Ablation of functional TRPV6 channels did not abolish the spontaneous Ca^2+^ oscillations, which depend on both Ca^2+^ release and Ca^2+^ influx, but significantly reduced their frequency in isolated primary MEECs (Fig. 4) as well as in complete uterine tissue (Fig. 5). Hennes et al. also observed spontaneous Ca^2+^ oscillations in MEECs, which they explained by the induction of mechanical stimulation of the tissue during experimental handlings prior to the fluorescent measurements [63]. While this might be a possible reason for Ca^2+^ oscillations seen in freshly isolated uterine tissue, it does not reasonably explain spontaneous Ca^2+^ signals in the isolated primary MEECs. The slightly higher basic level of cytosolic Ca^2+^ in wildtype MEECs (Fig. 4d), which might be due to a small Ca^2+^ influx via TRPV6, might maintain the frequency of cytosolic Ca^2+^ oscillations. These Ca^2+^ oscillations may be triggered by the cytosolic Ca^2+^ itself, e.g. via Ca^2+^-induced Ca^2+^ release and inositol 1,4,5-trisphosphate (InsP_3_) receptors [66]. We did not study the source of Ca^2+^ during the spontaneous cytosolic Ca^2+^ oscillations in more detail and the precise mechanism of such Ca^2+^ oscillations, especially in non-excitable cells, such as MEECs, is still not completely understood. However, our data suggest that both Ca^2+^ infux and Ca^2+^ release are contributing and functional TRPV6 channels are involved.

The embryo-maternal communication is of the utmost importance for the coordination of the implantation process: early embryo implantation is a complex event that requires an implantation-competent blastocyst and a receptive endometrium. Prior to implantation, the endometrial cells (EECs) sense the presence of the invading embryo and initiate a number of intracellular signals, including Ca^2+^ oscillations [63]. These oscillations are essential for preparing the uterine lining for embryo implantation by influencing gene expression, cell adhesion and the secretion of factors that support implantation. In particular, the EECs signal the underlying endometrial stromal cells to undergo decidualization. During this process, stromal cells differentiate into round, secretory, pseudo-epithelial cells that provide nutrients for the invading blastocyst and alter local immunity to allow for proper implantation [67, 68]. Dysregulation of the Ca^2+^ oscillations might lead to impaired embryo implantation and reduced fecundity. The exact molecular players underlying Ca^2+^ oscillations, the subsequent downstream signaling pathways and the biological effects of their activation are still unclear. Yet, we have no direct experimental evidence that the reduced fecundity of *Trpv6* KO mice is due to the altered Ca^2+^ oscillations in endometrial cells. However, the TRPV6 channel could possibly be a sensor and messenger molecule in the EECs involved in the transformation of extracellular stimuli into the influx of Ca^2+^, inducing and coordinating underlying signaling pathways. Our findings that *Trpv6*-deficient females have reduced Ca^2+^ oscillations in endometrial cells support this hypothesis.

It is still unclear whether TRPV6 plays a direct role as maternal endometrial sensor for the blastocyst signaling but considering that lack of functional TRPV6 channels results in impaired fecundity, it might be involved in the early implantation process. The loss of TRPV6-mediated Ca^2+^ entry could lead to a higher incidence of implantation failure, which would require multiple attempts to reach a successful mating, and thus to a longer latency to the first and subsequent pregnancies. Furthermore, Ca^2+^ influx via TRPV6 might be involved in the signaling that triggers decidualization of the underlying stromal cells. Decidual cells are important for embryo implantation and the maintenance of pregnancy. The observed smaller litter sizes from homozygous *Trpv6*-deficient mothers might be the result of implantation failure of some but not all embryos. Implantation could also be affected by the lack of TRPV6 in the trophoblasts of the blastocyst, which form an altered extracellular matrix [49]. *Trpv6* deletion affects Ca^2+^ signaling in MEECs and female fecundity to the same extend as the *Trpv6* pore mutant. Thus, as in the epididymis [1] the effects are mediated by the missing channel function and not by other functions of the protein.

Female mice expressing a non-functional TRPV6 channel protein (*Trpv6*^*mt/mt*^) revealed increased LH blood levels (Fig. 1e). This might represent a compensatory effect to a reduced TRPV6-dependent Ca^2+^ signaling. In this respect, estrogen has been shown to upregulate TRPV6 in MEECs [55]. However, LH directly increases the progesterone level, which might be the reason for a shorter estrous cycle as observed in the *Trpv6*^*mt/mt*^ mice (Fig. 1f). We found that although the time spent in estrus stage by the *Trpv6*^*mt/mt*^ females was not significantly different from that of the wild-type mice, there was a clear tendency for the *Trpv6*^*mt/mt*^ females to have a shorter estrus. We observed that while in *Trpv6*^*mt/mt*^ mice the estrous stage occurred almost exclusively during nighttime, wild-type mice were still in the estrous phase the next morning. A shorter estrus leads to a smaller time window for the receptivity of the uterus and for the implantation of the embryo and thus to a lower fecundity.

Our experiments revealed that the Ca^2+^ content of the CPA-sensitive intracellular Ca^2+^ stores was increased upon functional ablation of TRPV6. We did not study this in more detail, and we have no explanation for that yet. Maybe TRPV6 is expressed in intracellular organelles and contributes to a consistent leakage from these stores. However, since the frequency of Ca^2+^ oscillations, which also depend on intracellular Ca^2+^ release (see Fig. 5), are reduced in *Trpv6*-deficient cells, more Ca^2+^ might remain in the stores.

Since the pharmacological tools, including cis-22a which is the most effective antagonist available, have only been proven to affect Ca^2+^ influx and membrane currents of overexpressed TRPV6 rather than specifically targeting native TRPV6 function, we used MEECs from *Trpv6*^*−/−*^ and *Trpv6*^*mt/mt*^ animals as controls [1, 2], especially for electrophysiological measurements, rather than pursuing a pharmacological strategy. Years ago, we developed the *Trpv6*-deficient mouse models *Trpv6*^*−/−*^ and *Trpv6*^*mt/mt*^ as valid controls for wild-type animals, and we have used them ever since as controls for wild-type animals, as well as for organs and cells isolated from them. In our opinion, using a genetic approach to eliminate TRPV6 activity in order to control its cellular function in the wild-type may reduce the off-target effects typically associated with pharmacological approaches.

A low number of endometrial cells positive for GFP may reflect the rapid turnover of endometrial cells in the brief murine estrus cycle and the cycle-dependent TRPV6 expression. The genetic approach to label these cells is binary, requiring Cre-mediated recombination to switch on GFP expression. Thus, it may take up to 24 hours after initial TRPV6 expression to detect GFP. The Rosa26 locus is a genetic region used in many mouse lines to integrate transgenes and achieve consistent gene expression across different cell types and developmental stages. Although this Rosa26 locus is generally considered to be expressed ubiquitously, there are reports of reduced expression in certain cell types and variation in expression levels depending on the specific inserted genes [69, 70]. In addition to the cell type, specific transgene constructs or other regulatory influences could contribute to this phenomenon. The genetic background of the Rosa26 reporter mouse strains (C57BL/6J for eR26-τGFP and C57BL/6 N for eR26-GCaMP3) could also result in differences between MEECs positive for GCaMP3, which are isolated from *Trpv6*-IC/eR26-GCaMP3 animals, and MEECs positive for GFP, which are isolated from *Trpv6*-IC/eR26-τGFP animals. It is also known that the 3’ coding sequence is expressed at significantly lower levels than the 5’ coding sequence [71], but this hardly applies to the GFP and GCaMP3 reporter constructs used here.

Our study provides the first insight into the physiological function of TRPV6 channels in the mouse uterus and their potential importance for female fecundity and fertility. Whether TRPV6 is directly involved in signaling between the blastocyst and the endometrium, and thus in the implantation process itself is not yet known and requires further investigation. In this regard, an endometrial-specific deletion of the *Trpv6* gene would help to study the function of TRPV6 in endometrial epithelial cells in more detail. However, the results could lead to novel approaches to improve the treatment of women with reproductive disorders, such as those associated with defective Ca^2+^ regulation, either in vivo or for in vitro fertilization.

## Supplementary Information

Below is the link to the electronic supplementary material.

ESM 1 (DOCX 1.61 MB)

## Data Availability

The mass spectrometry proteomics data have been deposited to the ProteomeXchange Consortium via the PRIDE [72] partner repository with the dataset identifier PXD060418 and 10.6019/PXD060418. The data reported in this paper is available from the corresponding author upon reasonable request.

## Abbreviations

ACE: angiotensin converting enzyme
CPA: cyclopiazonic acid
DVF: divalent-free
eR26: enhanced-Rosa26
FSH: follicle-stimulating hormone
IC: IRES-Cre
iDISCO: immunolabeling-enabled three-dimensional imaging of solvent-cleared organs
IRES: internal ribosome entry site
IV: current-voltage relationship
KO: knock out
LH: luteinizing hormone
MEEC: mouse endometrial epithelial cell
MESC: mouse endometrial stromal cell
SOCE: store-operated Ca^2+^ entry
TRPV6: transient receptor potential vanilloid 6
