# Supplementary material for: TRPV6 channel function is involved in endometrial epithelial cell Ca2+ signaling and female mouse fecundity
Source: Cell Mol Life Sci. 2025 Oct 7;82(1):346. doi: 10.1007/s00018-025-05857-9 (PMC12504177; doi:10.1007/s00018-025-05857-9)
Supplement: Supplementary file 1 — (DOCX 1.61 MB) [file 18_2025_5857_MOESM1_ESM.docx]

***Supplementary Information***

**TRPV6 channel function is involved in endometrial epithelial cell Ca2+ signaling and female mouse fecundity**

Adela Sota*^,1^, Andreas Beck*^,1^, Philipp Wartenberg^1,2^, Anna-Lena Gehl^1^, Manuel Winter^1^, Ulrich Wissenbach^1^, Marc Freichel^3^, Markus R. Meyer^1^, Ulrich Boehm^1,2^, Veit Flockerzi^1^, Claudia Fecher-Trost^1^ and Petra Weissgerber^1^

* contributed equally

^1^ Experimental and Clinical Pharmacology and Toxicology, Center for Molecular Signaling (PZMS), Saarland University, 66421 Homburg, Germany.

^2^ Center for Gender-Specific Biology and Medicine (CGBM), Saarland University, 66421 Homburg, Germany

^3^ Institute of Pharmacology, Heidelberg University, 69120 Heidelberg, Germany

corresponding author: petra.weissgerber@uni-saarland.de

**Supplementary figures S1-S3**

**
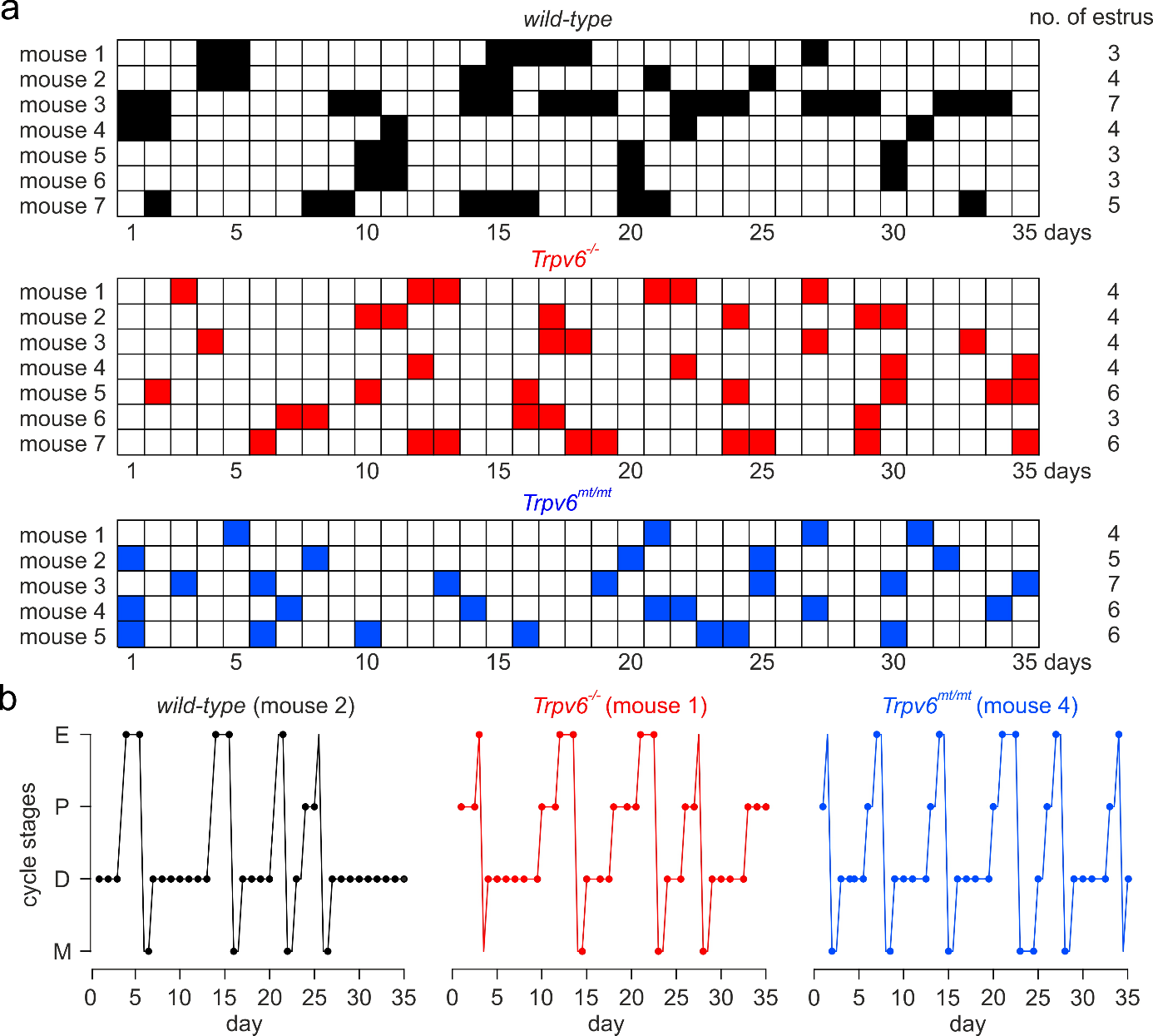
**

**Fig. S1 Estrous cycle in *wild-type*, *Trpv6^-^*^/-^ and *Trpv6*^mt/mt^ mice**. (**a**) Estrous cycles of 7 *wild-type* (black), 7 *Trpv6^-/-^* (red) and 5 *Trpv6^mt/mt^* (blue) mice throughout a period of 35 days. The filled boxes represent the days at estrus. (**b**) Representative estrous cycle throughout a period of 35 days for each genotype (E, estrus; P, proestrus; D, diestrus; M, metestrus).


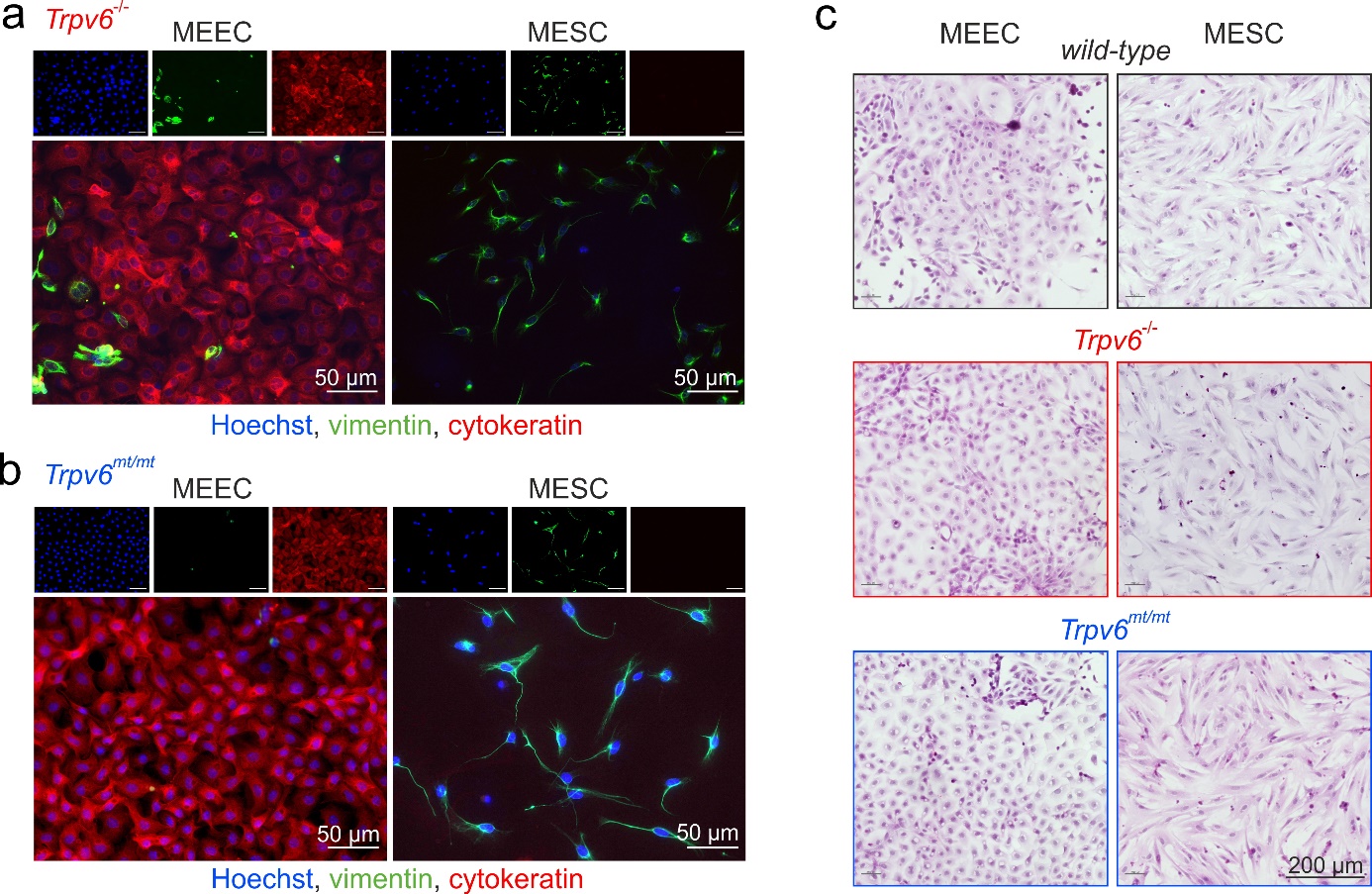


**Fig. S2 MEEC and MESC cultures from wild-type, *Trpv6^-/-^* and *Trpv6^mt/mt^* mice**. (**a**, **b**) Staining against cytokeratin (red) and vimentin (green) in MEEC and MESC cultures, isolated from *Trpv6^-/-^* (a) and *Trpv6^mt/mt^* (b) mice. Hoechst was used to stain nuclei (blue; top: single pictures; bottom: merged pictures). (**c**) Hematoxylin and eosin staining in MEECs and MESCs from *wild-type* (black), *Trpv6^-/-^* (red) and *Trpv6^mt/mt^* (blue) mice. Note that neither the MEEC nor the MESC cultures of the different genotypes reveal any apparent differences.


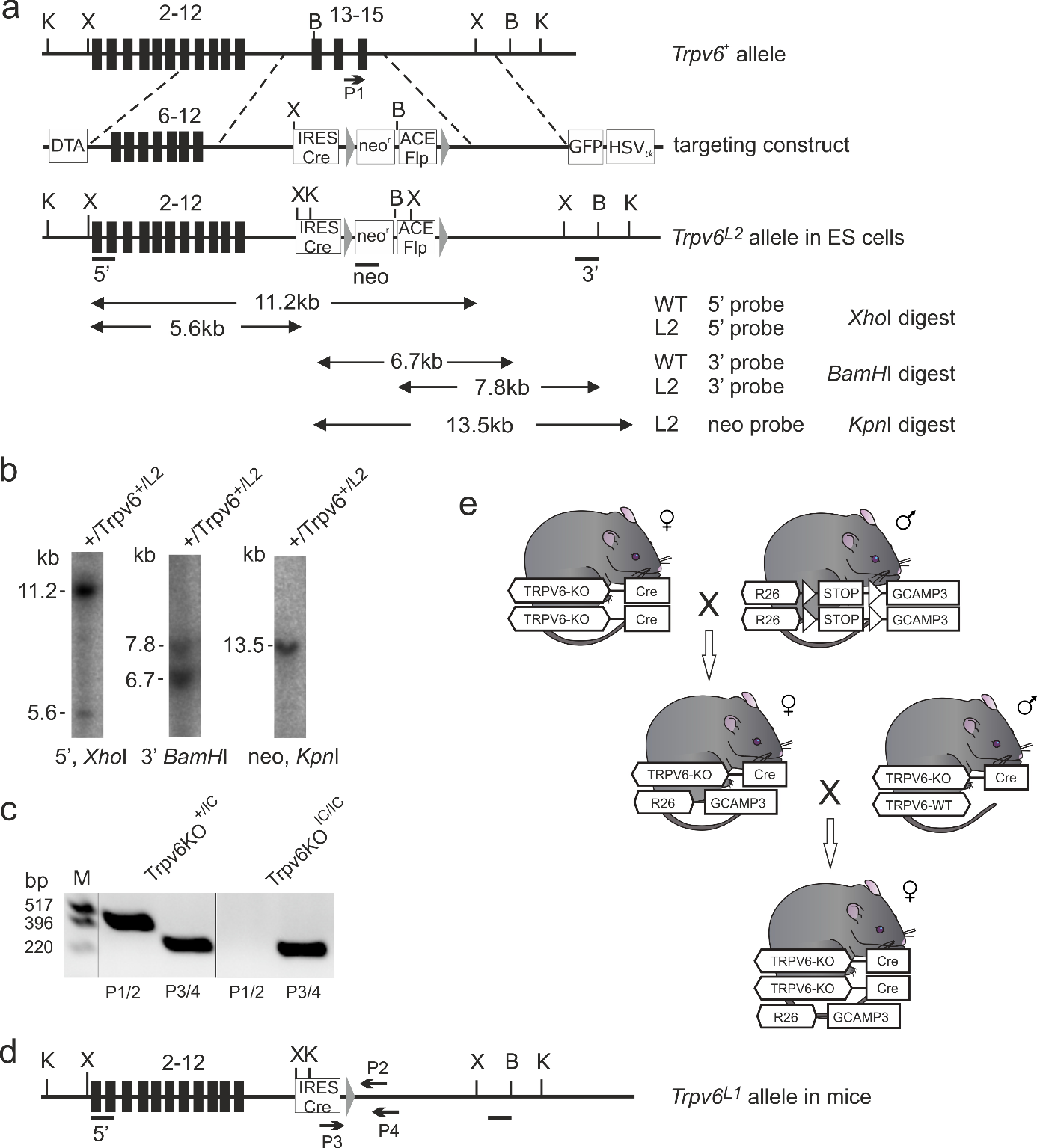


**Fig. S3 Targeting strategy for the generation of *Trpv6*^-/-^ (KO)-IC mice.** (**a**) *Trpv6* wild-type (*Trpv6^+^*) allele, targeting vector and *Trpv6*-L2 allele. Translated exons (not in scale) are represented as filled boxes. Exons 13-15 are removed in the targeting vector and exon 12 is followed by an internal ribosome entry site (IRES) accompanied by a Cre recombinase complementary DNA. A FRT site (grey triangles) -flanked PGK-neo Flp-ACE cassette is located downstream of the IRES-Cre cassette. Additionally, an enhanced GFP cDNA and the herpes simplex virus thymidine kinase (HSV_tk_) cassette were introduced 3´ and a diphtheria toxin A (DTA) cassette 5` for negative selection (X, *Xho*I; K, *Kpn*I; B, *BamH*I). Primer localization is indicated by arrows. Probes and sizes of genomic DNA fragments as expected by Southern Blot analysis: 5´prime probe (*XhoI* cut): *wt* 11.2 kb, L2 5.6 kb; 3´prime probe (*Bam*HI cut): *wt* 6.7 kb, L2 7.8 kb; internal neo probe (*Kpn*I cut): *wt* L2 13.5 kb. (**b**) Identification of correct homologous recombination in *Trpv6^+/L2^* via Southern Blot analysis. Probes and sizes of genomic DNA and PCR fragments are indicated. (**c**) Genotyping of heterozygous and homozygous *Trpv6KO^+/IC^* and *Trpv6KO^IC/IC^* via PCR. PCR fragment sizes for the identification of *Trpv6KO^+/IC^ and Trpv6KO^IC/IC^* mice: P1/P2 wt: 406 bp; *Trpv6*KO-IC P3/P4: 253 bp. (M, marker) (**d**) PCR strategy and primer (P3-4) localization for the identification of *Trpv6KO-IC* (*Trpv6^L1^* allele) mice. Fragment size see c. Small bar: 3´probe. (**e**) Breeding strategy to generate double transgenic *Trpv6*KO-IC/eR26-GCaMP3 females for further functional analyses. The *Trpv6KO-IC* line was bred to *Rosa26-*GCAMP3 reporter mice to enable endogenous GCAMP3-expression upon Cre-mediated recombination in cells with *Trpv6-*promotor activity.
